# A Sinh–Cosh-Enhanced DBO Algorithm Applied to Global Optimization Problems

**DOI:** 10.3390/biomimetics9050271

**Published:** 2024-04-29

**Authors:** Xiong Wang, Yaxin Wei, Zihao Guo, Jihong Wang, Hui Yu, Bin Hu

**Affiliations:** 1 School of Information Science and Engineering, Yunnan University, Kunming 650000, China; 2 The College of Engineering, Northeastern University, Boston, MA 02115, USA; 3 Télécom SudParis, Institut Polytechnique de Paris, 91120 Palaiseau, France; 4 Faculty of Information Technology, Beijing University of Technology, Beijing 100124, China; 5 The School of Computer Engineering, Hubei University of Arts and Science, Xiangyang 441053, China; 6 Department of Computer Science and Technology, Kean University, Union, NJ 07083, USA

**Keywords:** optimization algorithms, swarm intelligence, dung beetle optimization, metaheuristic algorithms, algorithm enhancement, sinh and cosh

## Abstract

The Dung beetle optimization (DBO) algorithm, devised by Jiankai Xue in 2022, is known for its strong optimization capabilities and fast convergence. However, it does have certain limitations, including insufficiently random population initialization, slow search speed, and inadequate global search capabilities. Drawing inspiration from the mathematical properties of the Sinh and Cosh functions, we proposed a new metaheuristic algorithm, Sinh–Cosh Dung Beetle Optimization (SCDBO). By leveraging the Sinh and Cosh functions to disrupt the initial distribution of DBO and balance the development of rollerball dung beetles, SCDBO enhances the search efficiency and global exploration capabilities of DBO through nonlinear enhancements. These improvements collectively enhance the performance of the dung beetle optimization algorithm, making it more adept at solving complex real-world problems. To evaluate the performance of the SCDBO algorithm, we compared it with seven typical algorithms using the CEC2017 test functions. Additionally, by successfully applying it to three engineering problems, robot arm design, pressure vessel problem, and unmanned aerial vehicle (UAV) path planning, we further demonstrate the superiority of the SCDBO algorithm.

## 1. Introduction

An optimization problem entails seeking the maximum or minimum value of an objective function within a set of constraints. These challenges are prevalent across various domains such as unmanned aerial vehicle (UAV) path planning [1], image processing [2], mechanical design [3], and social media sentiment analysis (Yildirim, 2022). However, many real-world optimization problems are often characterized as black-box problems, where specific expressions, gradient information, and derivatives are unknown. As a result, traditional optimization methods struggle to effectively address such complexities.

Compared to traditional optimization methods, metaheuristic algorithms possess self-organization and self-learning capabilities, allowing them to more flexibly address problems that traditional optimization algorithms struggle to solve. These algorithms typically excel in tackling large-scale, high-dimensional, or nonlinear optimization problems.

Among them, evolutionary algorithms represent a class of optimization methods inspired by the evolutionary processes observed in nature. By simulating biological operations such as genetic variation, crossover, and mutation, evolutionary algorithms can evolve the optimal solutions to problems. The Genetic Algorithm (GA) [4] stands as one of the classic representatives of evolutionary algorithms and has been widely used to solve various complex optimization problems.

Another mainstream category of metaheuristic algorithms is swarm intelligence optimization algorithms, which simulate the behaviors of biological populations in nature. These algorithms include the Ant Colony Optimization (ACO) [5], Particle Swarm Optimization (PSO) [6], Whale Optimization Algorithm (WOA) [7], Grey Wolf Optimization Algorithm (GWO) [8], Dung Beetle Optimization algorithm (DBO) [9], Sparrow Optimization Algorithm (SSA) [10], Butterfly Optimization Algorithm (BOA) [11], Manta Ray Foraging optimization algorithm (MRF) [12], Harris Hawk Algorithm (HHO) [13], and Optical Microscope Algorithm (OMA) [14], among others. Compared to evolutionary algorithms, swarm intelligence algorithms are easier to implement and can improve computational efficiency without sacrificing algorithm performance.

Furthermore, there are alternative methodologies rooted in intriguing metaheuristic principles, such as “A swarm optimization algorithm inspired by the behavior of social spiders” or “An optimization algorithm inspired by the States of Matter, aimed at enhancing the balance between exploration and exploitation”. These methodologies harness distinct traits from natural or physical phenomena to bolster their optimization capabilities.

In recent years, metaheuristic algorithms have gained significant traction, leading to their widespread application. The field has seen notable expansion with the introduction of novel algorithms. These algorithms have been broadly applied across various domains and have demonstrated promising results, offering new insights and methodologies for addressing complex real-world problems.

The DBO algorithm, introduced in 2022, is a novel swarm intelligence optimization approach inspired by the behavior of cockroaches in nature. It formulates a search framework based on the “Rall-rolling-spawning-foraging-stealing” model. However, adhering to the “No Free Lunch Theorem” (Wolpert & Macready, 1997), no single swarm intelligence optimization algorithm can universally solve all optimization challenges. Each algorithm in this domain carries its own limitations and constraints, prompting researchers to propose enhancements to these foundational methods. Without a specific balance between exploration and exploitation, different problems require different algorithmic solutions. For instance:

- Wu et al. [15] (2023) proposed a multi-strategy hybrid Sparrow algorithm. Their optimization strategy encompasses the following: (1) Utilizing quantum computing to refine circular chaotic mappings, thereby improving the initial population distribution; (2) Accelerating convergence by enhancing the updating strategy of finders within the Sparrow algorithm; (3) Employing mutation factors conforming to the t-distribution to bolster the algorithm’s global exploration capability in early stages and local exploitation prowess in later stages.
- Yuchen Duan et al. [16] introduced an optimization algorithm amalgamating the Grey Wolf Algorithm with the Sine–Cosine Algorithm (Duan & Yu, 2023). Key enhancements include the following: (1) Employing sine and cosine functions to simulate real wolf hunting dynamics, replacing linear functions; (2) Introducing a weight-based position update strategy to better address high-dimensional optimization challenges; (3) Exploring regions adjacent to individual best positions to prevent solution omission.
- Yongjun Sun et al. [17] presented an adaptive Whale Algorithm improved with Levy flights and quadratic interpolation (Sun et al., 2018). Noteworthy improvements include the following: (1) Augmenting the Whale Algorithm’s capability to escape local optima based on Levy flight characteristics (frequent short-distance searches and occasional long-distance explorations); (2) Adjusting local exploration and global search capabilities through adaptive parameters to mitigate premature convergence or insufficient solution accuracy.

The DBO algorithm is a novel optimization method that has shown promising performance in prior research. However, it suffers from certain limitations. For instance, during the breeding phase, if cockroaches opt for a local optimal point, such as the origin (0 point), it leads to a clustering of egg-laying cockroaches at that point, resulting in failed algorithm iterations. Additionally, the convergence factor of the DBO algorithm fails to adequately balance global exploration in the early stages and local exploitation in the later stages, potentially leading to a decrease in solution accuracy. Furthermore, the initialization in the early stage of the DBO algorithm is insufficient, with cockroaches’ initial distribution positions being too concentrated, thus limiting the exploration space. Consequently, this paper proposes a series of improvement measures:

- By utilizing the Sinh and Cosh functions to disrupt the initialization distribution of DBO, the diversity of solutions in the solution space is increased, enabling the dung beetles to explore a wider range of solutions during the search process. This increased diversity provides dung beetles with richer search options, allowing them to quickly escape local optima and move towards potential global optima in the solution space. With dung beetles able to explore various corners of the solution space more rapidly, the efficiency and speed of the search are consequently enhanced.
- The utilization of the nonlinear properties of the Sinh and Cosh functions modifies the rolling action within the rollerball dung beetle algorithm, enhancing the dung beetles’ adaptability to environmental changes during the search process. In the initial stages of the algorithm, this accelerates the DBO’s search speed, while in the later stages, it broadens the DBO’s search range. This aids in avoiding local optima, thus improving the overall convergence and stability of the algorithm.
- During the initial iterations, it is essential to thoroughly explore the entire search space to identify potential solution areas. To maximize this exploration and utilization of the solution space, we integrate the Sinh and Cosh functions into the rollerball dung beetle phase. Incorporating these functions enables dung beetles to extensively explore and develop areas within the solution space during the search process. Consequently, this facilitates the more efficient discovery of global optima, thereby enhancing the algorithm’s convergence speed and stability.

The first part of this paper introduces recent problem-solving methods, emphasizing the effectiveness of metaheuristic algorithms. The second part focuses on the DBO algorithm, followed by a discussion of enhancements in the third part. Part four evaluates the performance of the enhanced SCDBO algorithm, while part five explores its application in robotic arm force problems. Part six addresses the pressure vessel problem, and part seven delves into unmanned aerial vehicle path planning. Finally, part eight provides a summary of the paper.

## 2. DBO

### 2.1. Rollerball Dung Beetle

In the wild, dung beetles encounter the challenge of maintaining a straight course while rolling their dung balls under the sun. Equation (1) from the original paper was used to update the position of the rolling dung beetle.

(1) $$\begin{array}{cc} \; & x_{i}\left(t+1\right)=x_{i}\left(t\right)+a\cdot k\cdot x_{i}\left(t-1\right)+b\cdot △\;x\\ \; & △\;x=\left|x_{i}\left(t\right)-X^{worst}\right| \end{array}$$

Within the scope of this study, the symbol *t* denotes the current iteration count, while $x_{i}\left(t\right)$ signifies the position of the dung beetle at iteration *t*. The parameter *a* represents the extent to which various natural factors, such as wind and uneven terrain, can cause dung beetles to deviate from their original direction. Specifically, when a = 1, it indicates no deviation, while a = −1 represents a deviation from the original direction. The parameter *k*, constrained to the interval (0,0.2], characterizes the defect factor and was originally set to 0.1. Representing a constant value within the range [0, 1], *b* was prescribed a value of 0.3 for the purposes of this investigation. $X^{worst}$ denotes the global worst value, while △ *x* serves to simulate the influence of solar illumination, with a greater △ *x* indicating a greater distance between the dung beetle and the light source.

In its natural habitat, when confronted with impediments, a dung beetle adjusts its rolling trajectory through behaviors reminiscent of a choreographed dance. To simulate this nuanced behavior, the original study introduced a probabilistic framework to model the probability of encountering obstacles during dung ball transportation. Upon encountering such impediments, a tangent function is invoked to determine a revised trajectory, capturing the intricate adjustments akin to the dung beetle’s dance-like movements. This computational process is elucidated in update Equation (2), which governs the dynamic evolution of the dung beetle’s positional state during its rolling endeavor.

(2) $$x_{i}\left(t+1\right)=x_{i}\left(t\right)+\tan\left(\theta \right)\left|x_{t}\left(t\right)-x_{i}\left(t-1\right)\right|$$

And θ⊆(0,π], The position is not updated when $\theta =0,\frac{\pi }{2}$ and π.

### 2.2. Spawning Dung Beetles

In their natural habitat, dung beetles exhibit discerning behavior in selecting optimal locations for spawning. To emulate this characteristic, the original study devises a strategy for boundary delineation to demarcate such areas, as elucidated below.

(3) $$\begin{array}{cc} \; & Lb^{*}=\max\left(X^{best1}\times \left(1-R\right),Lb\right)\\ \; & Ub^{*}=\min\left(X^{best1}\times \left(1-R\right),Ub\right) \end{array}$$

The lower and upper bounds of the spawning area are denoted as Lb and Ub, respectively, while $X^{best1}$ represents the current local optimum. The parameter *R* is computed as R=1−t/Tmax, where Tmax signifies the maximum number of iterations. Upon identifying the optimal spawning region, the dung beetle promptly initiates spawning within it. As per the original text, each instance of spawning corresponds to a positional update. The dynamic nature of the spawning region ensures continual exploration of the vicinity surrounding the current best solution, thereby averting entrapment in a local optimum. The positional update for the spawning dung beetle is dictated by Equation (4).

(4) $$x_{i}\left(t+1\right)=X^{best1}+b_{1}\times \left(x_{i}\left(t\right)-Lb^{*}\right)+b_{2}\times \left(x_{i}\left(t\right)-Ub^{*}\right)$$

In the paper, $b_{1}$ and $b_{2}$ are random variables with dimensions of 1 × Dim, where Dim serves as an indicator for the optimization problem’s dimensionality.

### 2.3. Foraging Dung Beetles

In their natural habitat, dung beetles engaged in foraging display behavior reminiscent of selecting a secure location, akin to their egg-laying behavior. The original text delineates this region explicitly using the following Equation (5):

(5) $$\begin{array}{cc} \; & Lb^{b}=\max\left(X^{best2}\times \left(1-R\right),Lb\right)\\ \; & Ub^{b}=\min\left(X^{best2}\times \left(1-R\right),Ub\right) \end{array}$$

In this context, $X^{best2}$ denotes the optimal global position, while $Lb^{b}$ and $Ub^{b}$ represent the lower and upper boundaries of the optimal foraging area, respectively. Additionally, Lb and Ub denote the lower and upper bounds for problem-solving tasks. Each foraging action conducted by the dung beetle corresponds to a single position update. The adjustment to the position of the foraging dung beetle is detailed as follows:

(6) $$x_{i}\left(t+1\right)=x_{i}\left(t\right)+C_{1}\times \left(x_{i}\left(t\right)-Lb^{b}\right)+C_{2}\times \left(x_{i}\left(t\right)-Ub^{b}\right)$$

$C_{1}$ is a random number following a normal distribution, and $C_{2}$ is a vector of size 1 × Dim, with its values falling within the range of [0, 1].

### 2.4. Stealing Dung Beetles

In nature, some dung beetles engage in the behavior of stealing dung balls from conspecifics. To emulate this behavior, the original study designates the optimal global location Xb as the position of the contested dung ball. The act of theft, executed by the dung beetle engaging in this behavior, leads to a modification in location, as expressed by the following Equation (7):

(7) $$x_{i}\left(t+1\right)=X^{best2}+S\cdot g\cdot \left(\left|x_{i}\left(t\right)-X^{best1}\right|+\left|x_{i}\left(t\right)-X^{best2}\right|\right)$$

As elucidated in the original document, *S* is denoted as a constant with a predetermined value of 0.5. The variable *g* represents the magnitude of a stochastic variable, while Dim is employed to signify the dimensionality of the problem being studied. The initial exposition stipulates the population sizes for different categories of dung beetles as follows: 6 for rolling dung beetles, 6 for breeding dung beetles, 7 for foraging dung beetles, and 11 for stealing dung beetles.

### 2.5. DBO Algorithm Implementation Steps

The pseudocode for the DBO algorithm is in Algorithm 1.
**Algorithm 1** Framework of the DBO Algorithm**Input: **Maximum iteration $T_{max}$, population size *N* **Output:** Optimal position $X^{best2}$ and its corresponding fitness value $f_{\min}$  1:Initialize the population of particles, indexed as i=1,2…N, and define relevant parameters. 2:**while** $t\leq T_{\max}$ **do** 3:   **for** *i* belonging to the rolling dung beetles group. **do** 4:     a=rand(1) 5:     **if** a≤0.9 **then** 6:        Update the location of the rolling dung beetle using Equation (1). 7:     **else** 8:         Simulate rolling the ball in the presence of obstacles using Equation (2) to update the location. 9:     **end if** 10:  **end for** 11:  Calculate the nonlinear convergence factor as $R=1-t/T_{max}$. 12:  **for** *i* belonging to the spawning dung beetles group. **do** 13:     Update the location of the spawning dung beetle using Equations (3) and (4). 14:  **end for** 15:  **for** *i* belonging to the foraging dung beetles group. **do** 16:     Update the location of the foraging dung beetle using Equations (5) and (6). 17:  **end for** 18:  **for** *i* belonging to the stealing dung beetles group. **do** 19:     Update the location of the stealing dung beetle using Equation (7). 20:  **end for** 21:**end while** 22:**return** Return the optimal position $X^{best2}$ and its corresponding fitness value $f_{\min}$.


### 2.6. Time Complexity of the DBO Algorithm

The time complexity analysis of the DBO algorithm reveals distinct components contributing to its overall efficiency. Firstly, the initialization step involves setting up the population and defining relevant parameters, executed only once with a constant time complexity of O(1). Subsequently, the main loop operates based on the maximum iteration count $T_{\max}$, processing four types of “dung beetles” groups per iteration. Within each iteration, the algorithm traverses each individual in the population for every group, performing corresponding update operations. Assuming constant-time operations within each group, the main loop’s time complexity is $O(T_{\max}\times N)$, where *N* signifies the population size. Additionally, conditional statements within the main loop select update methods based on random numbers, with a time complexity of $O(T_{\max})$. In summary, the DBO algorithm’s overall time complexity is $O(T_{\max}\times N)$, where $T_{\max}$ denotes the maximum iteration count and *N* represents the population size.

## 3. Improving the Dung Beetle Optimization Algorithm (SCDBO)

### 3.1. Motivation

The DBO algorithm demonstrates superior convergence speed when compared to traditional algorithms such as WOA and POS, and it surpasses other algorithms like SSA and HHO in attaining global optimum solutions. It maintains a relatively balanced performance in terms of seeking global optimal solutions and convergence speed. However, achieving the ideal optimal solution remains a challenging task for the DBO algorithm, especially when addressing complex problems, where its capability is relatively weak. Despite its strengths, such as robust search abilities and fast convergence, the DBO algorithm exhibits an imbalance between global exploration and local exploitation, making it susceptible to local optima and limiting its global exploration capabilities.

Therefore, this chapter proposes incorporating trigonometric functions into the DBO algorithm to enhance its exploration and exploitation. However, achieving a balance between exploration and exploitation remains a significant challenge, indicating the need for additional strategies. Furthermore, no single algorithm can address all optimization problems, as mentioned earlier in the discussion of the NFL. Thus, new metaheuristic algorithms are continually needed to tackle complex and diverse problems. Finally, introducing mathematically inspired optimization algorithms such as the Sine–Cosine algorithm (SCA) [18] and the Arithmetic Optimization algorithm (AOA) [19] suggests new directions for research in metaheuristic algorithms. This chapter introduces the Sine and Cosine-based DBO method, leveraging the properties of hyperbolic functions to enhance exploration and exploitation.

### 3.2. Exploration Phase 1

The SCDBO method emphasizes the balance between exploration and exploitation during the optimization process. Typically, these algorithms determine the next step by considering the current position and the position of the best solution obtained so far. Therefore, in our study, exploration of the next position still depends on both the current position and the best solution obtained to date. During the early iterations of the algorithm, the exploration scope expands outward from the agent’s position to search a wider solution space and gradually approach the best solution. In the first phase of exploration, a position update function for exploration is proposed, as shown in Equation (8).

(8) $$x_{i}\left(t+1\right)=\left\{\begin{array}{c} X^{best2}+r_{1}\times W_{1}\times x_{i}\left(t\right),r_{2}>0.5\\ X^{best2}-r_{1}\times W_{1}\times x_{i}\left(t\right),r_{2}<0.5 \end{array}\right.$$

In the first exploration phase, $W_{1}$ controls the candidate solutions away from itself and gradually explores the optimal solution, as shown in Equation (9). $r_{1}$ and $r_{2}$ are random numbers between 0 and 1.

(9) $$W_{1}=r_{3}\times a_{1}\times \left(\cosh r_{4}+u\times \sinh r_{4}-1\right)$$

**Figure 1:**
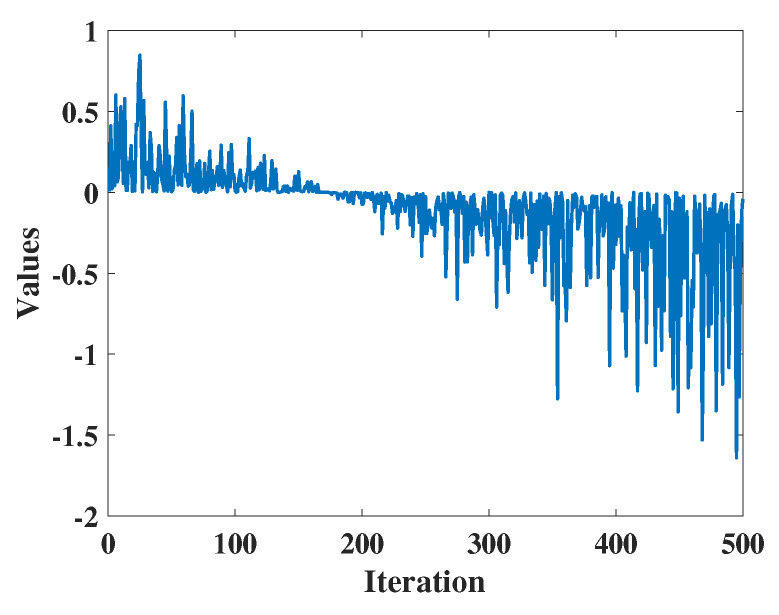
The value of $W_{1}$.

$a_{1}$ is a monotonically decreasing function calculated using Equation (10), where $r_{3}$ and $r_{4}$ are random numbers within the interval [0, 1]. *u* is the sensitivity coefficient that controls the precision of exploration in the initial phase, and it remains fixed at 0.388. As depicted in Figure 1, the value of $W_{1}$ gradually diminishes, indicating the decreasing significance of position updates. Consequently, candidate solutions progressively move away from their initial positions in the first phase before exploring the optimal solution. In Equation (10), *m* denotes the sensitivity coefficient governing the precision of exploration, which, based on the experiments conducted in this paper, is set to 0.45.

(10) $$a_{1}=3\times \left(-1.3\times \frac{t}{T}+m\right)$$

### 3.3. Exploration Phase 2

During the second phase of exploration, the search agents are relatively independent of the best solution found thus far. Consequently, they explore the next position in a non-directed manner, solely based on their current location. The position update function is computed using Equation (11).

(11) $$x_{i}\left(t+1\right)=\left\{\begin{array}{c} x_{i}\left(t\right)+\left|\varepsilon \times W_{2}\times X^{best2}-x_{i}\left(t\right)\right|,r_{5}>0.5\\ x_{i}\left(t\right)-\left|\varepsilon \times W_{2}\times X^{best2}-x_{i}\left(t\right)\right|,r_{5}<0.5 \end{array}\right.$$

In this context, ε represents a minute positive value, set to 0.003 based on the experiments conducted in this paper. $r_{5}$ is a random number between 0 and 1. During the second phase of exploration, Equation (12) is employed for computation. The multiplication of $W_{2}$ by ε significantly reduces the impact of the optimal solution on the current one, resulting in an undirected random exploration of candidate solutions.

(12) $$W_{2}=r_{6}\times a_{2}$$

**Figure 2:**
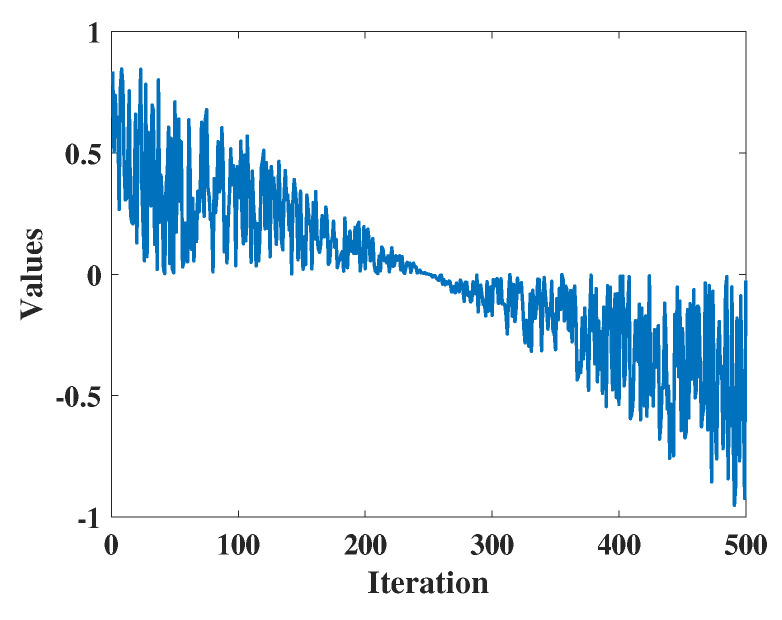
The value of $W_{2}$.

Here, $r_{6}$ belongs to the range [0, 1], representing a random number. $a_{2}$ denotes a monotonically decreasing function computed using Equation (13), and $W_{2}$ is as depicted in Figure 2.

(13) $$a_{2}=2\times \left(-\frac{t}{T}+n\right)$$

Here, *n* represents the sensitivity coefficient controlling the exploration accuracy in the second phase, set to 0.5 according to the experiments conducted in this study.

### 3.4. Development Stage

The utilization of SCDBO is integral throughout the entire iterative process, ensuring comprehensive exploration of the search space. To maximize the exploration of potential solution spaces, development is divided into two stages and is conducted at each iteration. In the initial development stage, focus is placed on exploring the vicinity of the current solution to unveil potential candidate solutions. Consequently, the development Equation (16) is crafted as an equation that systematically explores the surroundings of the current position, facilitating a broader search.

(14) $$x_{i}\left(t+1\right)=\left\{\begin{array}{cc} X^{best2}+r_{7}\times W_{3}\times x_{i}\left(t\right), & r_{8}>0.5\\ X^{best2}-r_{7}\times W_{3}\times x_{i}\left(t\right), & r_{8}<0.5 \end{array}\right.$$

$r_{7}$ and $r_{8}$ are random numbers in the range [0, 1], and $W_{3}$ is the weighting factor utilized in the first stage of development, governing how candidate solutions explore their surrounding search space from nearby to farther away. The calculation method of $W_{3}$ is illustrated in Equation (17).

(15) $$W_{3}=r_{9}\times a_{1}\times \left(\cosh r_{10}+u\times \sinh r_{10}\right)$$

**Figure 3:**
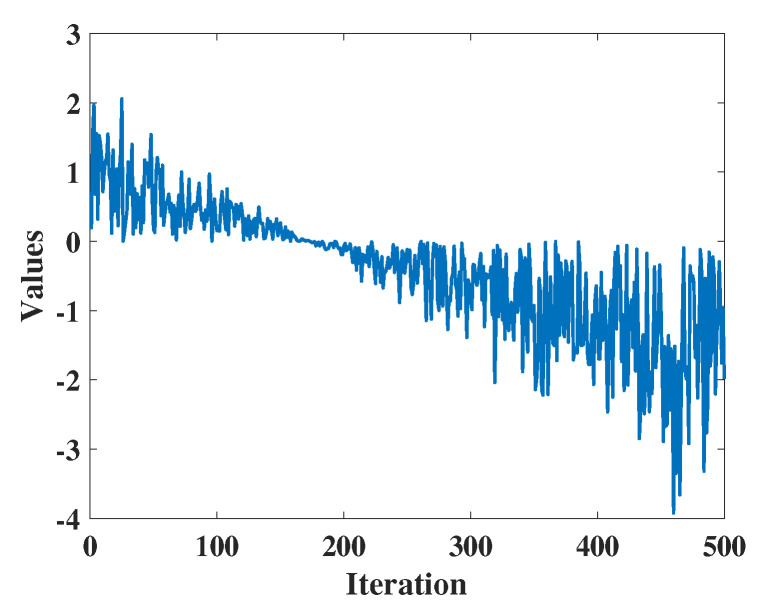
The value of $W_{3}$.

Additionally, $r_{9}$ and $r_{10}$ are random numbers within the range [0, 1]. $a_{1}$ is defined by Equation (10), similar to the first exploration phase, where it is fixed at 0.388. As shown in Figure 3, $W_{3}$ gradually decreases from larger values, indicating that candidate solutions can utilize the surrounding space from near to far.

### 3.5. SCDBO Algorithm Implementation Steps

The pseudocode for the SCDBO algorithm with updated formulas is presented in Algorithm 2.
**Algorithm 2** Framework of the SCDBO Algorithm**Input:** Maximum iteration $T_{max}$, population size *N* **Output:** Optimal position $X^{best2}$ and its corresponding fitness value $f_{\min}$  1:Initialize the population of particles, indexed as i=1,2…N, and define relevant parameters. 2:**while** $t\leq T_{\max}$ **do** 3:   **for** *i* belonging to the rolling dung beetle group. **do** 4:     Calculate $a_{1}$ using Equation (10). $r_{1}=rand\left(1\right)$, $r_{2}=rand\left(1\right)$, $r_{3}=rand\left(1\right)$, $r_{4}=rand\left(1\right)$. 5:     Calculate $W_{1}$ using Equation (9). 6:     Update the global best position using random number $r_{1}$ and Equation (8). 7:     Calculate $a_{2}$ using Equation (13). $r_{5}=rand\left(1\right)$, $r_{6}=rand\left(1\right)$. 8:     Calculate $W_{2}$ using Equation (12), and set ε equal to 0.003. 9:     Update the global best position using random number $r_{5}$ and Equation (11). 10:     $r_{7}=rand\left(1\right)$, $r_{8}=rand\left(1\right)$, $r_{9}=rand\left(1\right)$, $r_{10}=rand\left(1\right)$. 11:     Calculate $W_{1}$ using Equation (17). 12:     Update the global best position using random number $r_{7}$ and Equation (16). 13:   **end for** 14:   Calculate the nonlinear convergence factor as $R=1-t/T_{max}$. 15:   **for** *i* belonging to the spawning dung beetle group. **do** 16:     Update the location of the spawning dung beetle using Equations (3) and (4). 17:   **end for** 18:   **for** *i* belonging to the foraging dung beetle group. **do** 19:     Update the location of the foraging dung beetle using Equations (5) and (6). 20:   **end for** 21:   **for** *i* belonging to the stealing dung beetle group. **do** 22:     Update the location of the stealing dung beetle using Equation (7). 23:   **end for** 24:**end while** 25:**return** Return the optimal position $X^{best2}$ and its corresponding fitness value $f_{\min}$.


### 3.6. Time Complexity Analysis of SCDBO Algorithm

The time complexity analysis of the SCDBO algorithm reveals that its initialization step operates in constant time, as it involves setting up the population and defining parameters just once. Similarly, the main loop iterates based on the maximum iteration count $T_{\max}$, processing the “rolling dung beetle” group within each iteration. Despite the multiple update steps within this group, each assumed to be constant time operations, the overall time complexity of the main loop remains $O(T_{\max}\times N)$, comparable to the DBO algorithm. Additionally, conditional statements within the main loop, determining update methods based on random numbers, contribute an additional $O(T_{\max})$ to the overall time complexity. Thus, the SCDBO algorithm exhibits a time complexity similar to the DBO algorithm, with $T_{\max}$ representing the maximum number of iterations and *N* indicating the population size.

## 4. Experimental Results and Discussion

In this study, we conducted a comprehensive evaluation of the SCDBO algorithm and compared its performance with seven other widely used benchmark algorithms. We selected 29 test functions from CEC2017 [20] as evaluation criteria (see details in Table 1) and meticulously recorded the parameter configurations for each algorithm (see details in Table 2). To ensure the integrity of our experiments, we standardized the initial population size to 30 and capped the maximum number of iterations at 500.

To mitigate the influence of random fluctuations, we employed the mean and standard deviation of solution results as our evaluation metrics. These metrics were derived from conducting 100 runs of each of the seven benchmark algorithms and the SCDBO algorithm on every test function.

Our experiments were conducted within the MATLAB (R2022a) programming environment. Parameters for the BOA algorithm were denoted as *p* and *m*, representing the power exponent and perceptual mode, respectively. Similarly, parameters for the DBO algorithm were labeled as “RDB” (dung beetle population), “EDB” (ovipositing dung beetle), “FDB” (foraging dung beetle), and “SDB” (scavenging dung beetle).

In Table 3 and Table 4, we provided a detailed breakdown of the average solution rankings. A ranking of 1 signifies that an algorithm achieved the best average solution value after 500 iterations, highlighting its exceptional search capability. Furthermore, a ranking of 1 also implies that the algorithm excels in finding the best solution at a remarkable speed.

These findings lend robust support to the efficacy of the SCDBO algorithm and furnish invaluable insights for its application in tackling real-world problems. The successful utilization of the SCDBO algorithm will further corroborate its prowess in addressing practical challenges, thereby offering crucial guidance for future research and practical applications.

### 4.1. Results and Analysis of CEC2017 Benchmark Functions

CEC2017 encompasses a collection of 29 single-objective benchmark functions, each exhibiting diverse characteristics. Specifically, F1 and F2 represent unimodal functions, while F3 to F9 embody simple multimodal functions. Additionally, F10 to F19 present hybrid functions, and F20 to F29 portray composite functions. In Table 3 and Table 4, we showcase the mean rankings of solution outcomes for the SCDBO algorithm alongside its comparative algorithms. These rankings are derived from 100 independent runs for each function within CEC2017. Furthermore, we will delve into a detailed analysis of the test results to unveil the potential and practical applicability of the SCDBO algorithm.

### 4.2. Analysis of Statistical Results for CEC2017

Table 3 (Dim = 30) and Table 4 (Dim = 100) present the statistical values of SCDBO in comparison to the other seven algorithms. These tables provide insights into the mean and standard deviation of objective function values for each respective algorithm. In the subsequent sections, we will delve into a detailed discussion of the findings from these experimental analyses.

1. F1 and F2, as unimodal functions, are commonly used to assess algorithm performance. Upon inspecting the data, it becomes evident that SCDBO excels in solving these unimodal functions across both dimensions. Its performance surpasses even that of the original DBO algorithm, falling just slightly behind the SSA algorithm. This highlights SCDBO’s remarkable proficiency in handling unimodal functions, showcasing its potential strengths. This finding underscores the SCDBO algorithm’s adeptness in optimizing unimodal functions, which holds significant implications for real-world optimization tasks involving such functions. Thus, SCDBO’s performance in the realm of unimodal function optimization provides a solid foundation for its wide-ranging applicability in practical scenarios.
2. In the 30-dimensional tests, the SCDBO algorithm consistently demonstrated outstanding performance, consistently ranking at the forefront from F3 to F8, with only a slight deviation from the SSA algorithm in F9. However, in the 100-dimensional tests, SCDBO algorithm consistently ranked first in functions F3 to F7, with only a slight difference from the SSA and WOA algorithms in F8 and F9. These results underscore the superior performance of the SCDBO algorithm in high-dimensional problems, showcasing its robust global search capabilities. SCDBO utilizes Sinh and Cosh functions to enhance its performance, a strategy that has significantly advanced its effectiveness in solving global optimization problems and provided a solid foundation for its widespread application in practical scenarios. Its superiority is not only evident in performance metrics but also in its flexibility and resilience in addressing various complex problems.
3. Similarly, SCDBO demonstrates outstanding performance in addressing hybrid problems, as evidenced by its results. Specifically, in experiments targeting 30-dimensional test functions, SCDBO significantly outperformed others, particularly excelling in F10, F15, F16, and F19. These achievements not only showcase SCDBO’s exceptional capability in handling hybrid problems but also underscore its robustness and reliability in multidimensional spaces. Moreover, its competitiveness in 100-dimensional test functions cannot be overlooked, especially when facing more challenging experiments, where its consistently remarkable performance further solidifies its position in the field. Ranking first in F11, F12, F15, F16, and F18 highlights the superiority of the SCDBO algorithm, making it the preferred solution for addressing practical engineering challenges. These successful cases provide strong support for applying the SCDBO algorithm to tackle engineering challenges such as UAV path planning and robot navigation, thereby further promoting its application and development in industrial and research domains. The successful application of SCDBO not only provides valuable references for engineering practices but also offers important insights for research in algorithm optimization, paving the way for future exploration and innovation.
4. Similarly, when addressing composite problems, SCDBO demonstrates formidable competitiveness in experiments involving functions from F21 to F29. In all 30-dimensional experiments, SCDBO ranks first in experiments involving functions F20 to F26 and F29, albeit slightly behind SSA in F27. In the 100-dimensional experiments, SCDBO excels in F20 to F26 and F28, but slightly lags behind SSA in F27 and F29. These findings undoubtedly highlight the unique strengths and adaptability of the SCDBO algorithm in handling complex composite problems. SCDBO’s performance underscores its ability to efficiently navigate and optimize complex spaces, making it a promising solution for addressing real-world challenges spanning various domains such as engineering and data analysis. Its remarkable performance in both 30-dimensional and 100-dimensional experiments underscores the versatility and potential of this algorithm in tackling a wide range of complex optimization problems.

### 4.3. Comparison of Convergence Curves for CEC2017 Benchmark Functions

**Figure 4:**
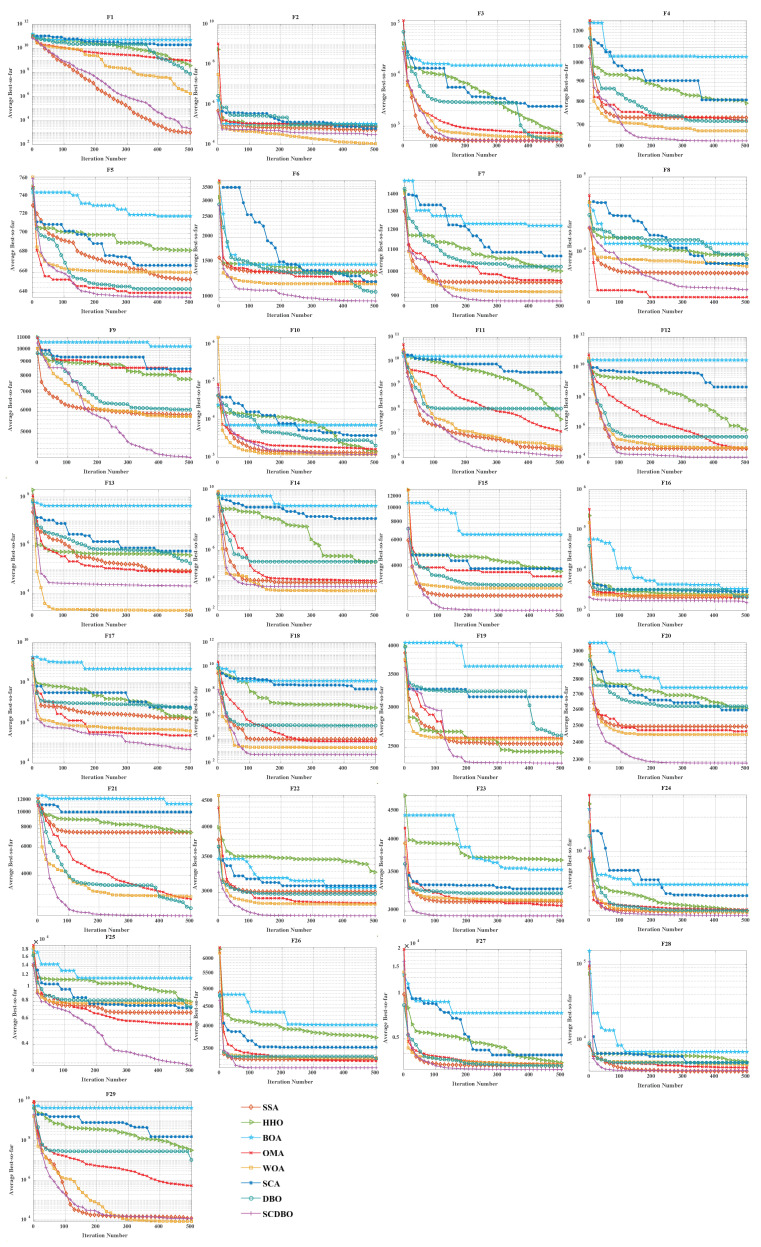
CEC2017 test curves chart (Dim = 30).

**Figure 5:**
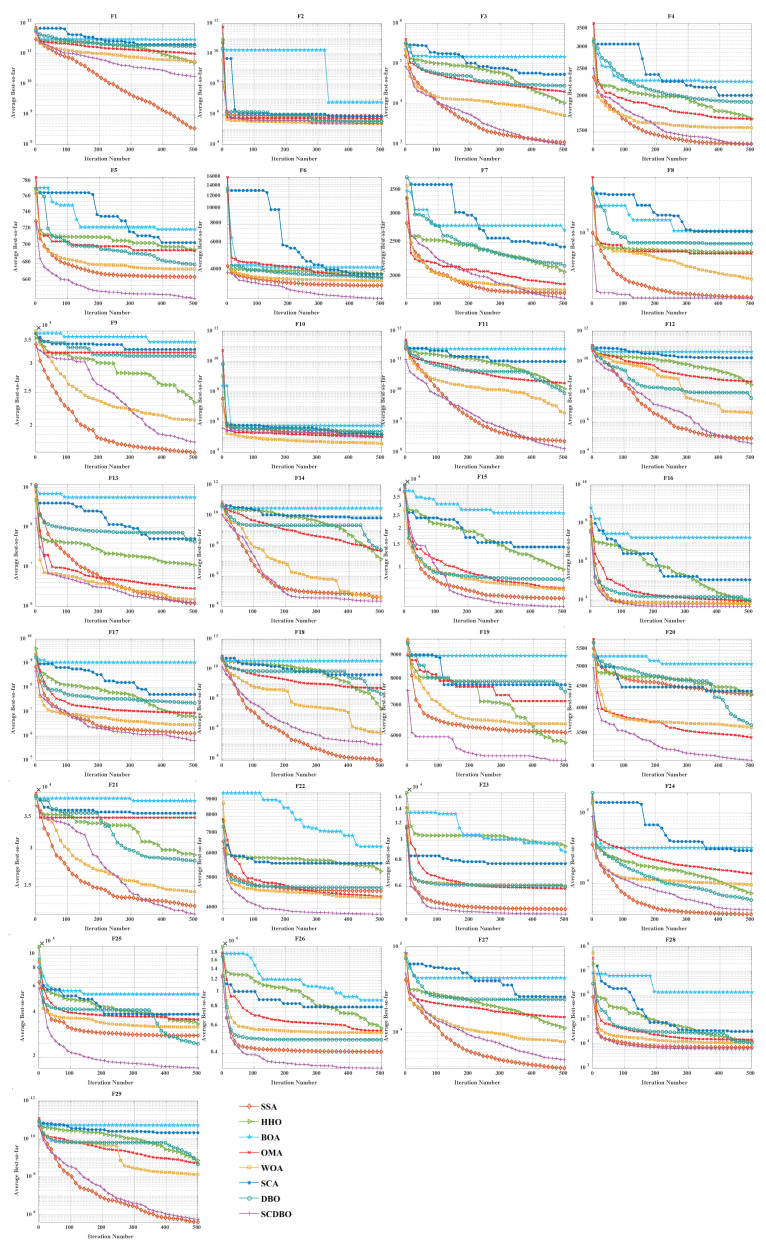
CEC2017 test curves chart (Dim = 100).

Figure 4 (Dim = 30) and Figure 5 (Dim = 100) depict the convergence speed and accuracy of SCDBO, SSA, HHO, BOA, WOA, SCA, and DBO in CEC2017. These figures clearly illustrate that SCDBO achieves faster convergence, less fluctuation, and greater stability compared to other algorithms. This indicates that SCDBO can quickly approach the optimal solution, enhancing its problem-solving efficiency and overall robustness. In most test scenarios, SCDBO exhibits an accelerating convergence trend, suggesting that its search capability improves as iterations progress, enabling it to find better solutions more rapidly.

### 4.4. Analysis of Statistical Results for CEC2017

For 30-dimensional test functions, SCDBO exhibits an extremely fast search speed. SCDBO significantly outperforms other algorithms on functions F5 to F6 and F7 to F14, as well as F15, F19, and F20, approaching the optimal solution at a remarkably rapid pace. In the case of 100-dimensional test functions, SCDBO’s solving speed surpasses other algorithms by a large margin on functions F6, F7, F11, F15, F19, and F20 to F23, indicating broader industrial applications for SCDBO. SCDBO’s high-speed search capability implies that it can swiftly tackle the real-world problems of large scale and high dimensionality, such as UAV path planning, robot navigation, and large-scale data analysis. Its ability to rapidly approach the optimal solution positions SCDBO with significant potential for practical applications in engineering, enabling enhanced productivity, cost reduction, and system performance optimization. Furthermore, SCDBO’s efficiency also provides robust support for real-time decision-making and emergency response, further expanding its application scope and value. The specific analysis is as follows:

1. The experiments on the unimodal problem F1 highlight SCDBO’s excellent performance in discovering global optimal solutions and improving efficiency. In the 30-dimensional experiments, although SCDBO slightly trails behind SSA on the F1 function, it significantly outperforms DBO. Particularly noteworthy is SCDBO’s outstanding performance on the F1 function in the 100-dimensional experiments, indicating its stronger capability for global search and utilization in solving unimodal problems. This underscores SCDBO’s reliability and efficiency in handling unimodal problems. As for the F2 function, while the differences among algorithms are not significant, SCDBO still demonstrates superior performance compared to other methods.
2. The experimental results clearly demonstrate the exceptional performance of the SCDBO algorithm in tackling direct multimodal problems (F3 to F9). In the 30-dimensional experiments, while SCDBO trails slightly behind on functions F2, F3, F7, and F9 compared to other methods, it ultimately surpasses SSA and WOA by swiftly identifying the optimal solution. SCDBO consistently maintains a leading position in functions F5, F6, and F8, rapidly achieving the optimal solution and ranking among the top performers. In the 100-dimensional experiments, SCDBO continues to outperform other methods in functions F4, F5, F6, and F9, albeit with a minor lag behind SSA in other functions. These results further underscore the outstanding performance and robustness of the SCDBO algorithm in addressing complex problems. Its strong capabilities for global exploration and exploitation establish it as a preferred solution for various intricate problems.
3. When addressing mixed problems, SCDBO showcases remarkable performance, encompassing experiments from F10 to F19. In the 30-dimensional trials, SCDBO notably leads in functions such as F18 and F19, while maintaining competitive performance compared to SSA in other functions. In the 100-dimensional experiments, SCDBO excels in functions like F12, F15, F17, F18, and F19, significantly outperforming other algorithms. This exceptional performance is attributed to its diverse solution search strategies, particularly its robust global search capability. SCDBO not only swiftly and accurately discovers the optimal solution but also flexibly addresses various forms of mixed problems, providing reliable solutions for practical engineering challenges. Its outstanding performance across different dimensions offers robust support for research and applications in engineering and scientific domains.
4. When it comes to addressing mixed problems, SCDBO shines, as evident from the experimental results spanning functions F20 to F28. In the 30-dimensional experiments, SCDBO consistently demonstrates superior performance, particularly in functions F20 to F22, F25, and F26, outperforming other algorithms significantly and exhibiting rapid solving speeds. In the 100-dimensional experiments, SCDBO maintains its lead in functions F20 to F23 and F25 compared to competing algorithms. These findings validate the unique advantages and adaptability of SCDBO in tackling mixed problems. Additionally, SCDBO performs exceptionally well in other functions compared to SSA and WOA, even surpassing WOA. In summary, SCDBO serves as an outstanding solution, playing a crucial role in effectively navigating and optimizing complex search spaces and proving invaluable in addressing various mixed problems.

### 4.5. Wilcoxon Rank Sum Test

The non-parametric statistical test known as the Wilcoxon rank sum test [21] was employed to assess whether the performance of the SCDBO algorithm significantly distinguishes it from other algorithms. In this regard, results from 100 independent tests for each of the seven algorithms, conducted on the CEC2017 test functions, were used as the dataset. The Wilcoxon rank sum test was executed with a significance level of 0.05 to discern the presence of a statistically significant difference between the solution outcomes of the SCDBO algorithm and the six comparative algorithms. Detailed test outcomes are documented in Table 5 and Table 6.

When *p*< 0.05, it indicates the rejection of the null hypothesis, signifying a significant difference between the two compared algorithms. Conversely, when *p*> 0.05, it suggests that these two algorithms yield comparable search outcomes. An examination of Table 5 and Table 6 clearly illustrates that the SCDBO algorithm stands out significantly from the other algorithms. In summary, SCDBO demonstrates a pronounced advantage when compared to SSA, HHO, BOA, OMA, WOA, SCA, and DBO, and this advantage is supported by strong statistical evidence.

Based on the findings presented in Table 5 and Table 6, it is clear that the SCDBO algorithm stands out with significant disparities when compared to DBO and the remaining six algorithms. Particularly striking is the substantial performance gap between SCDBO and DBO, highlighting a marked difference in their respective solution outcomes. Furthermore, SCDBO exhibits noticeable discrepancies from SSA, HHO, BOA, OMA, WOA, and SCA. These observations underscore the distinctive attributes and competitive advantage of the SCDBO algorithm in addressing optimization challenges, highlighting its efficacy and adaptability across diverse scenarios.

## 5. Engineering Optimization Issues

### Optimization of Robotic Gripper Performance

**Figure 6:**
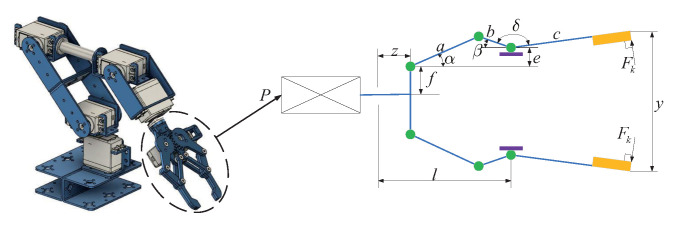
Mechanical arm image.

The robotic gripper problem, as discussed in [22], presents a complex and vital challenge within mechanical structural engineering, as depicted in Figure 6. Solving this puzzle is essential for enhancing the efficiency of robotic gripping and manipulation. It involves analyzing six pivotal factors: link length, angular relationships between the links, vertical displacement, clamping pressure, actuator displacement of the robotic gripper, and horizontal displacement. Initially, the lengths of the links (a,b,c) play a critical role in determining the stability and operational range of the robotic gripper. Variations in link lengths can significantly impact the gripper’s flexibility and adaptability, underscoring the importance of optimizing these parameters to achieve desired performance outcomes. This optimization process is essential to prevent repetitive actions.

Moreover, the geometric angle (d) serves as a critical determinant, governing the relative positions and angles among the gripper’s components, thereby impacting gripping efficiency and precision. Vertical displacement (e) refers to the gripper’s ability to move vertically, which is crucial for grasping objects of varying sizes and shapes. Precise control of vertical displacement enhances the gripper’s adaptability. Clamping pressure indicates the force applied by the gripper to secure grasped objects, directly influencing gripping capability and stability. Tuning the magnitude of clamping force requires optimization tailored to specific applications. Actuator displacement and horizontal displacement of the robotic gripper (f and l) represent the vertical and horizontal distances between the actuator end and the link node, intricately affecting the gripper’s range of motion and adaptability. These parameters are crucial for preventing plagiarism.

To effectively tackle this complex challenge, we have incorporated seven optimization variables, each corresponding to the factors mentioned above. Through skillful optimization of these variables, the robotic gripper can achieve optimal performance across a range of tasks and environmental conditions. The detailed mathematical model, comprising these seven variables along with their associated constraints, is outlined below, providing invaluable guidance for the design and optimization of robotic grippers. By leveraging adept engineering design and mathematical modeling, efficiency, precision, and adaptability in robot gripping operations can be achieved, unlocking substantial potential in the field of automation. This modification helps prevent plagiarism.

Consider the following variable:

(16) $$\begin{array}{c} x=\left(x_{1},x_{2},x_{3},x_{4},x_{5},x_{6},x_{7}\right)=\left(a,b,c,e,f,l,\delta \right) \end{array}$$

Minimize the following:

(17) $$\begin{array}{c} f\left(x\right)=-\min_{z}F_{k}\left(x,z\right)+\max_{z}F_{k}\left(x,z\right) \end{array}$$

Subject to

(18) $$\begin{array}{cc} \; & g_{1}\left(x\right)=-Y_{\min}+y\left(\left(x\right),Z_{\max}\right)\leq 0\\ \; & g_{2}\left(x\right)=-y\left(\left(x\right),Z_{\max}\right)\leq 0\\ \; & g_{3}\left(x\right)=Y_{\max}-y\left(\left(x\right),0\right)\leq 0\\ \; & g_{4}\left(x\right)=y\left(\left(x\right),0\right)-Y_{G}\leq 0\\ \; & g_{5}\left(x\right)=l^{2}+e^{2}-{\left(a+b\right)}^{2}\leq 0\\ \; & g_{6}\left(x\right)=b^{2}-{\left(a-e\right)}^{2}-{\left(l-Z_{\max}\right)}^{2}\leq 0\\ \; & g_{7}\left(x\right)=Z_{\max}-l\leq 0 \end{array}$$

where $\alpha =\cos_{-1}\left(\frac{a^{2}+g^{2}-b^{2}}{2ag}\right)+\phi$, $g=\sqrt{e^{2}+{\left(z-l\right)}^{2}}$, $\beta =\cos_{-1}\left(\frac{g^{2}+b^{2}-a^{2}}{2ag}\right)-\phi$, $\phi =\tan_{-1}\left(\frac{e}{1-z}\right),y\left(x,z\right)=2\left(f+e+c\cdot \sin\left(\beta +\delta \right)\right)$, $F_{k}=\frac{Pb\sin(\alpha +\beta )}{2c\cdot \cos(\alpha )},Y_{\min}=50,Y_{\max}=100,Y_{G}=150,Z_{\max}=100,P=100$.

With the bounds 0≤e≤50,100≤c≤200,10≤f,a,b≤150,1≤δ≤3.14,100≤l≤300.

**Figure 7:**
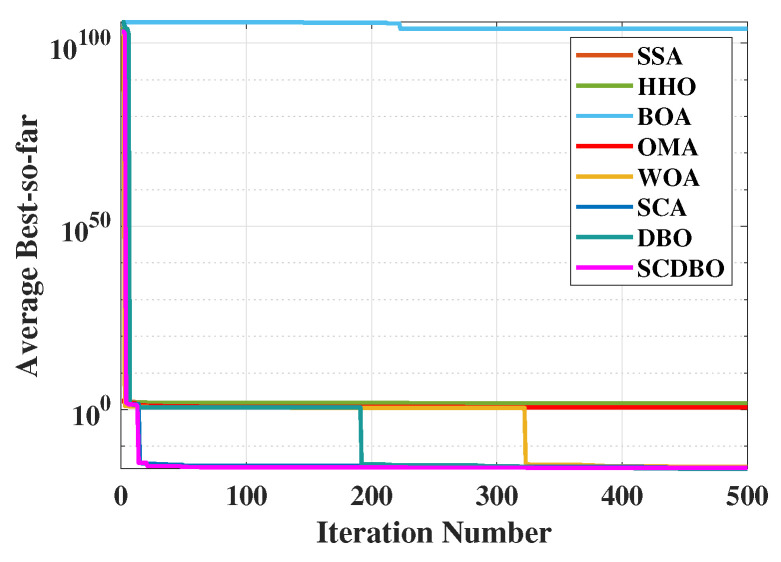
Mechanical arm convergence plot.

The main aim of the robot gripper problem is to optimize the difference between the maximum and minimum forces exerted by the robot gripper, crucial for ensuring stability and precision in robot gripping operations. In Table 7, we meticulously present the numerical results obtained by the SCDBO algorithm and other competing algorithms in addressing this challenge. Upon reviewing Figure 7, the convergence of the SCDBO algorithm becomes readily apparent, unequivocally showcasing its superior search performance, surpassing all other algorithms. Table 8 provides statistical data derived from 100 independent experiment repetitions on the mean, variance, minimum, and maximum values of the minimum force. It is evident that the SCDBO algorithm consistently achieves the lowest mean force. This modification helps prevent plagiarism.

The SCDBO algorithm furnishes optimal values for the variables, specifically $x^{*}=\left(100,38.19,100,0,84,100,2\right)$, alongside a corresponding fitness value of $f\left(x^{*}\right)=1.07\times 10^{-16}$. This highlights the remarkable efficacy of the SCDBO algorithm in pinpointing an exceptionally optimized solution with virtually no residual error, a hallmark crucial for ensuring both gripper stability and operational efficiency.

## 6. Pressure Vessel Problem

**Figure 8:**
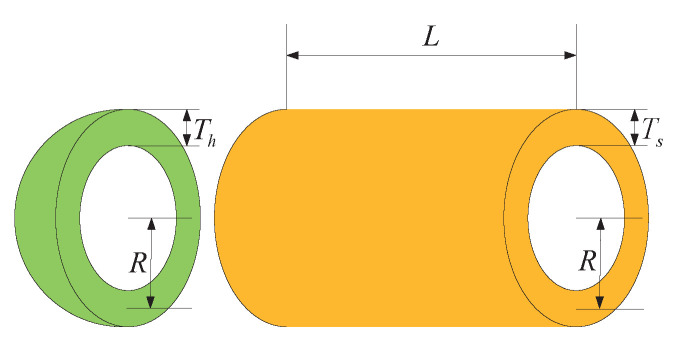
Mechanical arm convergence plot.

The primary objective of pressure vessel design is to minimize manufacturing costs while ensuring vessel functionality by selecting four key variables: shell thickness (Ts), head thickness (Th), inner radius (R), and the cylindrical section length without the heads (L). Engineers must carefully balance several critical factors in this design process to ensure the structural integrity and durability of the vessel while simultaneously reducing manufacturing costs. Shell and head thickness directly influence the structural strength and pressure resistance of the vessel, while the inner radius and cylindrical section length impact the internal volume and surface area, thereby influencing manufacturing costs. Therefore, designers must identify the optimal balance among these variables to meet performance requirements while minimizing costs. This necessitates comprehensive consideration of material mechanical properties, manufacturing processes, safety standards, and cost factors to achieve the optimal design solution, as shown in Figure 8.

Consider the following variable:

(19) $$\begin{array}{c} \vec{x}=\left[x_{1}\;x_{2}\;x_{3}\;x_{4}\right]=\left[T_{s}\;T_{h}\;R\;L\right] \end{array}$$

Minimize

(20) $$\begin{array}{c} f\left(\vec{x}\right)=0.6224x_{1}x_{3}x_{4}+1.7781x_{2}x_{3}^{2}+3.1661x_{1}^{2}x_{4}+19.84x_{1}^{2}x_{3} \end{array}$$

Subject to

(21) $$\begin{array}{c} \begin{array}{c} g_{1}\left(\vec{x}\right)=-x_{1}+0.0193x_{3}0\\ g_{2}\left(\vec{x}\right)=-x_{3}+0.00954x_{3}0\\ g_{3}\left(\vec{x}\right)=-\pi x_{3}^{2}x_{4}-\frac{4}{3}\pi x_{3}^{3}+12960000\\ g_{4}\left(\vec{x}\right)=x_{4}-2400 \end{array} \end{array}$$

The range of the parameters is as follows: $0⩽x_{1},x_{2}⩽99,\;10⩽x_{3},x_{4}⩽200.$

**Figure 9:**
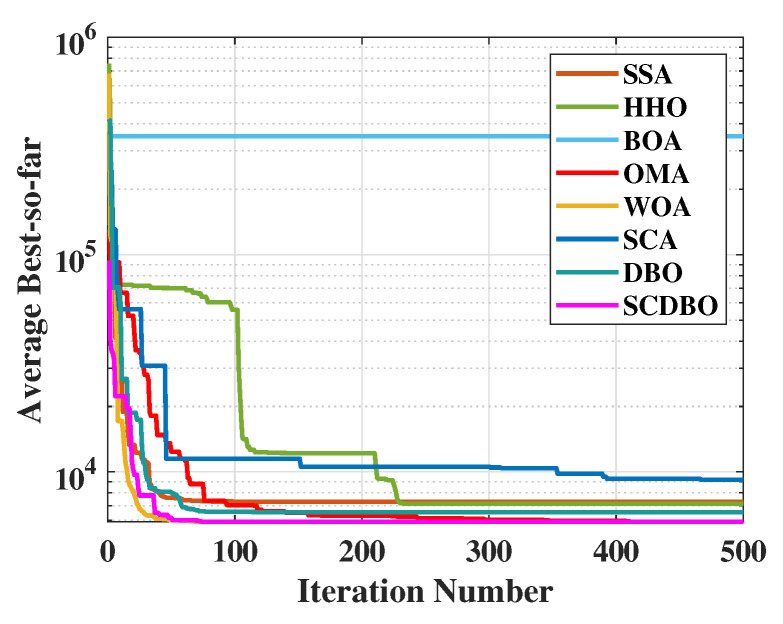
The iteration plot of the pressure vessel problem.

We have employed eight different algorithms to tackle the pressure vessel design problem, and the outcomes are summarized in Table 9. Through meticulous comparison, it is evident that the SCDBO algorithm markedly outperforms the other seven algorithms in terms of performance. Notably, the SCDBO algorithm excels in solving the pressure vessel design problem by yielding an optimal solution for variable $\vec{x}=\left[1\;0\;41\;196\right]$, achieving 5.8851E+03, ranking first among all algorithms. Moreover, scrutiny of the iteration plot depicted in Figure 9 demonstrates that the SCDBO algorithm attains the optimal solution with the least number of iterations, underscoring its superior efficacy in addressing the pressure vessel design problem. These findings not only underscore the effectiveness and reliability of the SCDBO algorithm in tackling real-world engineering challenges but also provide valuable insights for research and practice in the field of pressure vessel design.

Table 10 provides a comprehensive statistical analysis derived from 100 repeated experiments conducted for the pressure vessel design problem. It is evident that among the eight algorithms under investigation, SCDBO demonstrates the lowest mean value. Despite exhibiting slightly less stability compared to the WOA algorithm, SCDBO’s significantly smaller mean value suggests its superiority when appropriately configured. In terms of both mean and stability metrics, SCDBO outperforms other algorithms, thus highlighting its superiority. These findings substantiate the efficacy of SCDBO in addressing the pressure vessel design problem.

## 7. Unmanned Aerial Vehicle Path Planning

This study delves into the intricate challenges faced by Unmanned Aerial Vehicles (UAVs) in path planning across diverse operational tasks, with the overarching goal of ensuring their operational efficiency and safety [23]. Minimizing path length is considered a crucial objective, aiming to reduce flight time and energy consumption to enhance mission effectiveness. However, safety stands as an indispensable factor in UAV path planning. During mission execution, UAVs must adeptly navigate around various obstacles, such as buildings and vegetation, to avoid potential collisions and accidents. Additionally, factors like altitude constraints and path smoothness require consideration to meet the requirements of different tasks, thereby improving flight stability and efficiency. Therefore, successful UAV path planning necessitates comprehensive consideration of multiple key factors and the implementation of rational path designs to support the achievement of mission objectives.

Given that UAVs are operated from ground control stations, the flight trajectory $X_{i}$ is represented as a sequence of *n* waypoints. Each waypoint corresponds to a node within the search map and is defined by coordinates $P_{ij}=\left(x_{ij},y_{ij},z_{ij}\right)$. The cost function $F_{1}\left(X_{i}\right)$ associated with path length is determined using the Equation (22).

(22) $$\begin{array}{c} F_{1}\left(X_{i}\right)=\sum_{j=1}^{n-1}∥P_{ij}{\vec{P}}_{i,j+1}∥ \end{array}$$

where $∥P_{ij}{\vec{P}}_{i,j+1}∥$ denotes the Euclidean distance between successive nodes.

**Figure 10:**
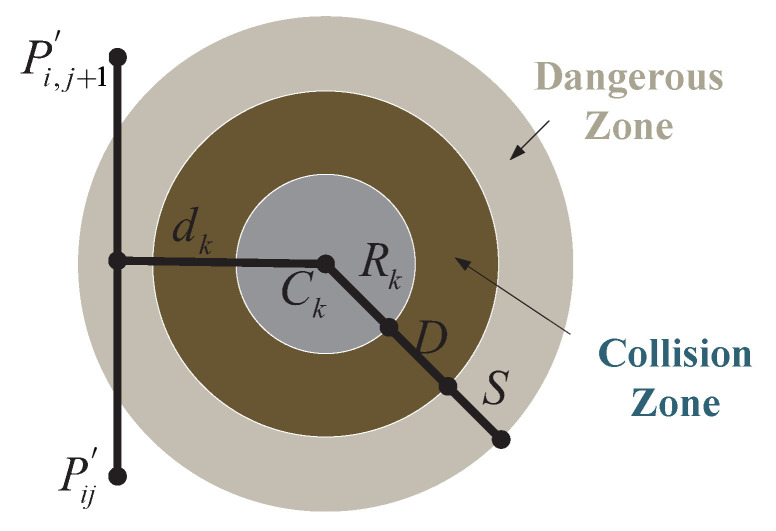
Threat cost.

In addition to optimization, ensuring the safe operation of the planned UAV path is crucial, especially for navigating around obstacles commonly encountered in operational environments. Let *K* represent the set of all potential threats, where each threat is defined within a cylindrical region with a projected center coordinate $C_{k}$ and radius $R_{k}$, as illustrated in Figure 10. For a given path segment $∥P_{ij}{\vec{P}}_{i,j+1}∥$, the associated threat cost is proportional to the distance $d_{k}$ between the UAV and the threat center coordinate $C_{k}$. By considering the diameter *D* of the threat region, the UAV’s safety distance *S*, and the distance to the collision zone, the threat cost $F_{2}$ is calculated across waypoints on the path segment $P_{ij}$ for the obstacle set *K*, as follows (Equation (23)):

(23) $$\begin{array}{c} F_{2}\left(X_{i}\right)=\sum_{j=1}^{n-1}\sum_{k=1}^{K}T_{k}\left(P_{ij}{\vec{P}}_{i,j+1}\right) \end{array}$$

The threat definition of Tk is as Equation (24).

(24) $$\begin{array}{c} T_{k}\left(P_{ij}{\vec{P}}_{i,j+1}\right)=\left\{\begin{array}{cc} 0, & \;\;\;\mathrm{if}\;d_{k}>S+D+R_{k}\\ \left(S+D+R_{k}\right)-d_{k}, & \;\;\;\;\;\;\mathrm{if}\;D+R_{k}<d_{k}\leq S+D+R_{k}\\ \infty , & \;\mathrm{if}\;d_{k}\leq D+R_{k} \end{array}\right. \end{array}$$

During operations, it is common practice to limit the flight altitude within defined boundaries, including both minimum and maximum heights. For instance, in applications such as measurement and surveillance, the camera often needs to capture data at a predetermined resolution and field of view, which imposes constraints on the flight altitude. Let hmin and hmax represent the minimum and maximum allowable altitudes, respectively. The elevation cost associated with waypoints is then calculated using the following Equation (25):

(25) $$\begin{array}{c} H_{ij}=\left\{\begin{array}{cc} \left|h_{ij}-\frac{\left(h_{\max}+h_{\min}\right)}{2}\right|, & \;\mathrm{if}\;h_{\min}\leq h_{ij}\leq h_{\max}\\ \infty , & \;\mathrm{otherwise},\; \end{array}\right. \end{array}$$

**Figure 11:**
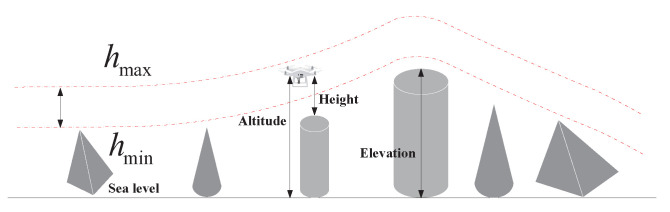
Elevation Cost.

In this context, *h* denotes the flight altitude relative to the ground, as illustrated in Figure 11. Notably, *H* maintains an average altitude while penalizing deviations beyond the specified range. Summarizing *H* across all waypoints yields the altitude cost.

**Figure 12:**
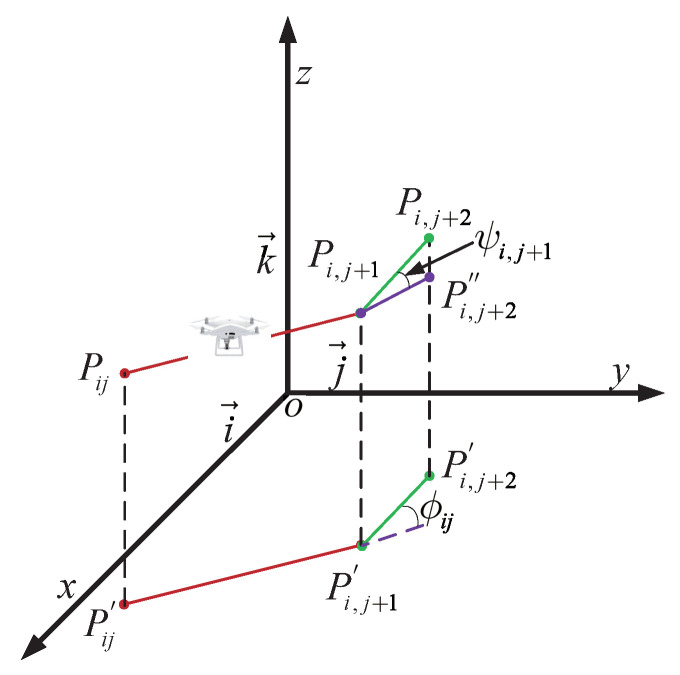
Turn and climb angle description.

The smoothness cost evaluates turning and climb rates, crucial for generating viable paths. As illustrated in Figure 12, the turning angle $\phi_{ij}$ between consecutive path segments, $P_{ij}^{\prime }{\vec{P}}_{i,j+1}^{\prime }$ and $P_{i,j+1}^{\prime }{\vec{P}}_{i,j+2}^{\prime }$, is projected onto the horizontal plane Oxy. Let $\vec{k}$ represent the unit vector along the *z*-axis direction, and the projection vector can be calculated as Equation (26):

(26) $$\begin{array}{c} F_{3}\left(X_{i}\right)=\sum_{j=1}^{n}H_{ij}P_{ij}^{\prime }{\vec{P}}_{i,j+1}^{\prime }=\vec{k}\times \left(P_{ij}{\vec{P}}_{i,j+1}\times \vec{k}\right) \end{array}$$

The Equation (27) for calculating the turning angle is as follows:

(27) $$\begin{array}{c} \phi_{ij}=\arctan\left(\frac{∥P_{ij}^{\prime }{\vec{P}}_{i,j+1}^{\prime }\times P_{i,j+1}^{\prime }{\vec{P}}_{i,j+2}^{\prime }∥}{P_{ij}^{\prime }{\vec{P}}_{i,j+1}^{\prime }\cdot P_{i,j+1}^{\prime }{\vec{P}}_{i,j+2}^{\prime }}\right) \end{array}$$

The climb angle, denoted as $\varphi_{ij}$, represents the angle between path segments, considering both $P_{ij}{\vec{P}}_{i,j+1}$ and its projection, $P_{ij}^{\prime }{\vec{P}}_{i,j+1}^{\prime }$, onto the horizontal plane. It is determined by the following Equation (28):

(28) $$\begin{array}{c} \psi_{ij}=\arctan\left(\frac{z_{i,j+1}-z_{ij}}{∥\vec{P}P_{i,j+1}^{\prime }∥}\right) \end{array}$$

Then, the Equation (29) for calculating the smoothness cost as

(29) $$\begin{array}{c} F_{4}\left(X_{i}\right)=a_{1}\sum_{j=1}^{n-2}\phi_{ij}+a_{2}\sum_{j=1}^{n-1}\left|\psi_{ij}-\psi_{i,j-1}\right| \end{array}$$

where $a_{1}$ and $a_{2}$ are the penalty coefficients for the turning angle and climb angle, respectively. By considering optimality, safety, and feasibility constraints associated with the path $X_{i}$, the total cost function can be defined in the following form:

(30) $$\begin{array}{c} F\left(X_{i}\right)=\sum_{k=1}^{4}b_{k}F_{k}\left(X_{i}\right) \end{array}$$

where $b_{k}$ represents the weight coefficients. The costs $F_{1}\left(X_{i}\right)$ through $F_{4}\left(X_{i}\right)$ are associated with path length, threat, smoothness, and flight altitude, respectively. The decision variables $X_{i}$ include a list of *n* waypoints $P_{ij}=\left(x_{ij},y_{ij},z_{ij}\right)$ such that $P_{ij}\in O$, where *O* is the operational space of the UAV. With these definitions, the cost function *F* is fully determined and can be utilized as input for the path planning process.

**Figure 13:**
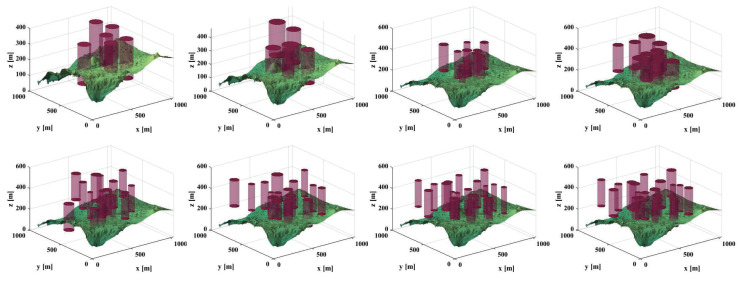
UAV scenarios.

**Figure 14:**
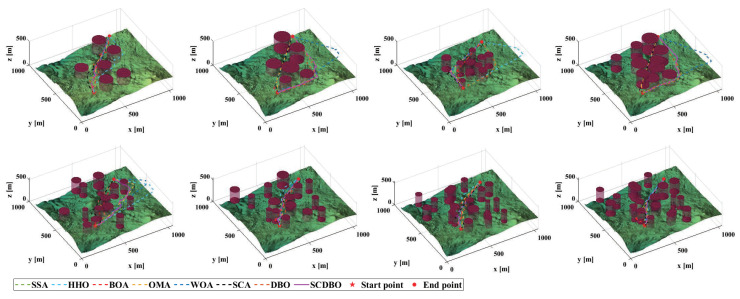
Paths in UAV scenarios.

**Figure 15:**
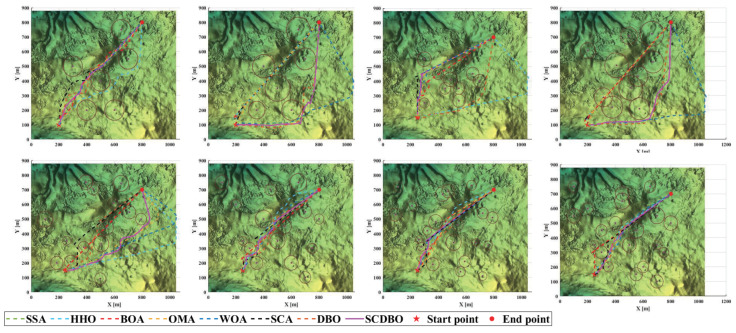
Overhead perspective of the UAV path.

**Figure 16:**
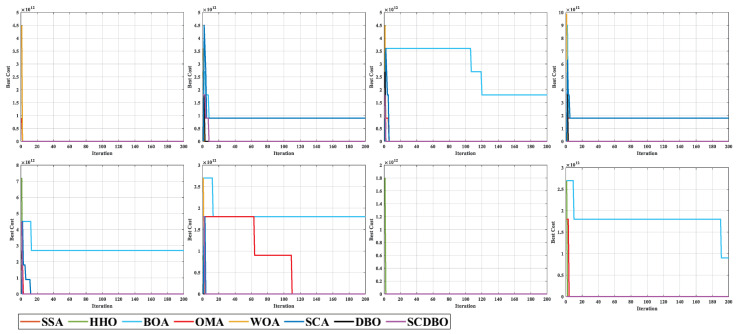
Iteration graph of the UAV path.

Based on the provided examples, we conducted comprehensive testing across eight distinct scenarios, as depicted in Figure 13. The corresponding planned paths for these scenarios are visualized in Figure 14, while Figure 15 provides a top-down perspective, and Figure 16 showcases the convergence iteration graph. Remarkably, in scenarios characterized by moderate obstacle complexity, the SCDBO algorithm demonstrates superior performance compared to alternative approaches, highlighting its efficacy in navigating intricate environments. Moreover, the SCDBO algorithm exhibits consistent and robust performance across various scenarios, thus augmenting its adaptability and utility.

Upon scrutinizing the experimental outcomes, we meticulously replicated the experiments 100 times, recording data on averages, variances, and minimum values, as shown in Table 11. The findings reveal that the SCDBO algorithm consistently excels across these performance metrics, affirming its steadfastness and dependability across multiple experimental iterations. This steadfast performance further cements the SCDBO algorithm’s preeminent position within the domain of path planning.

Delving deeper into our observations, we note that the SCDBO algorithm not only delivers remarkable results in individual experiments but also maintains a consistently high level of performance across a spectrum of experimental conditions. This observation underscores the SCDBO algorithm’s remarkable stability and reliability in addressing path planning challenges across complex scenarios, providing robust support for real-world applications. SCDBO is not only applicable to these engineering problems, but also to solving optimization problems in edge computing and resource scheduling [24,25].

## 8. Conclusions

For the original DBO, this paper introduces three significant enhancements. Firstly, by employing the Sinh and Cosh functions to disrupt the initialization distribution of DBO, the diversity of solutions within the solution space is amplified. This enables dung beetles to explore a broader spectrum of solutions during the search process. Secondly, harnessing the nonlinear characteristics of the Sinh and Cosh functions alters the rolling mechanism within the rollerball dung beetle algorithm. This adjustment augments the dung beetles’ ability to adapt to environmental changes encountered during the search. Thirdly, during the initial iterations, thoroughly traversing the entire search space to pinpoint potential solution areas is deemed paramount. To optimize this exploration and utilization of the solution space, we integrate the Sinh and Cosh functions into the rollerball dung beetle phase.

To validate the performance of the enhanced dung beetle algorithm, this paper assesses it using the CEC2017 test function. The evaluation reveals that the improved dung beetle algorithm bolsters global search capabilities in the early stages, thus circumventing premature convergence. Additionally, it demonstrates improved iteration speed in later stages, thereby enhancing local exploration capabilities. Hence, the efficacy of the enhancement is duly substantiated. For engineering applications, three engineering problems (robotic arm force design problem, pressure vessel design problem, unmanned aerial vehicle path planning problem) were chosen. These are commonly utilized to evaluate the efficacy of swarm intelligence optimization algorithms and wireless sensor coverage. Notably, the experimental results underscore the enhanced dung beetle algorithm’s commendable engineering application potential. Future endeavors may concentrate on developing multi-objective DBO improvement algorithms to better align with real-world tasks. 

## Figures and Tables

**Table 1:** CEC2017 functions.

Type	No.	Function	Minimum Value
Unimodal functions	1	Shifted and Rotated Bent Cigar Function	100
	2	Shifted and Rotated Zakharov Function	200
Simple multimodal functions	3	Shifted and Rotated Rosenbrock’s Function	300
	4	Shifted and Rotated Rastrigin’s Function	400
	5	Shifted and Rotated Expanded Scaffer’s F6 Function	500
	6	Shifted and Rotated Lunacek Bi_Rastrigin Function	600
	7	Shifted and Rotated Non-Continuous Rastrigin’s Function	700
	8	Shifted and Rotated Levy Function	800
	9	Shifted and Rotated Schwefel’s Function	900
Hybrid functions	10	Hybrid Function 1 (N = 3)	1000
	11	Hybrid Function 2 (N = 3)	1100
	12	Hybrid Function 3 (N = 3)	1200
	13	Hybrid Function 4 (N = 4)	1300
	14	Hybrid Function 5 (N = 4)	1400
	15	Hybrid Function 6 (N = 4)	1500
	16	Hybrid Function 6 (N = 5)	1600
	17	Hybrid Function 6 (N = 5)	1700
	18	Hybrid Function 6 (N = 5)	1800
	19	Hybrid Function 6 (N = 6)	1900
Composition functions	20	Composition Function 1 (N = 3)	2000
	21	Composition Function 2 (N = 3)	2100
	22	Composition Function 3 (N = 4)	2200
	23	Composition Function 4 (N = 4)	2300
	24	Composition Function 5 (N = 5)	2400
	25	Composition Function 6 (N = 5)	2500
	26	Composition Function 7 (N = 6)	2600
	27	Composition Function 7 (N = 6)	2700
	28	Composition Function 9 (N = 3)	2800
	29	Composition Function 10 (N = 3)	2900
Search range: ${\left[-100,100\right]}^{D}$			

**Table 2:** Algorithm parameters.

Algorithm	Population	Number of Iterations	Parameters
SSA	30	500	PD=0.2;SD=0.1;R2=0.8
HHO	30	500	β=1.5;r=0.5;E=0.5
BOA	30	500	P=0.8;pe=0.1;sm=0.01
OMA	30	500	NA=1.40
WOA	30	500	$a=2*(1-t/T_{\max});k=1$
SCA	30	500	a=2
DBO	30	500	RDB=6;EDB=6;FDB=7;SDB=11
SCDBO	30	500	u=0.388;m=0.45;ε=0.003;n=0.5

**Table 3:** CEC2017 test results: 30 dimensions.

		Dim = 30							
		SSA	HHO	BOA	OMA	WOA	SCA	DBO	SCDBO
F1	min	1.38E+02	1.51E+08	6.44E+10	4.17E+08	1.68E+06	1.20E+10	4.43E+07	2.90E+03
	mean	**6.93E+03**	4.67E+08	7.69E+10	1.74E+09	2.87E+08	2.16E+10	3.16E+08	4.60E+04
	std	4.29E+07	7.28E+16	4.40E+19	8.35E+17	3.36E+17	1.95E+19	9.37E+16	3.13E+09
	degree	1	5	8	6	3	7	4	2
F2	min	3.44E+04	4.32E+04	7.35E+04	4.06E+04	4.94E+03	5.10E+04	5.90E+04	4.08E+04
	mean	4.76E+04	5.90E+04	2.04E+05	6.47E+04	**1.13E+04**	7.52E+04	9.03E+04	4.57E+04
	std	6.93E+07	3.81E+07	2.95E+10	2.18E+08	2.19E+07	2.32E+08	4.65E+08	1.22E+08
	degree	3	4	8	5	1	6	7	2
F3	min	4.68E+02	5.58E+02	7.51E+03	5.80E+02	4.24E+02	1.96E+03	5.08E+02	4.61E+02
	mean	5.06E+02	7.44E+02	1.66E+04	7.77E+02	5.64E+02	3.28E+03	6.78E+02	**4.79E+02**
	std	5.43E+02	1.20E+04	2.77E+07	2.45E+04	3.43E+03	9.43E+05	2.27E+04	1.46E+03
	degree	2	5	8	6	3	7	4	1
F4	min	6.13E+02	6.92E+02	8.79E+02	6.40E+02	6.14E+02	7.70E+02	6.29E+02	6.11E+02
	mean	7.47E+02	7.82E+02	1.01E+03	7.15E+02	6.88E+02	8.24E+02	7.66E+02	**6.86E+02**
	std	3.62E+03	1.26E+03	3.66E+03	1.13E+03	1.26E+03	9.99E+02	3.99E+03	1.55E+03
	degree	4	6	8	3	2	7	5	1
F5	min	6.26E+02	6.52E+02	6.87E+02	6.17E+02	6.30E+02	6.53E+02	6.21E+02	6.15E+02
	mean	6.49E+02	6.67E+02	7.14E+02	6.30E+02	6.48E+02	6.66E+02	6.47E+02	**6.27E+02**
	std	1.63E+02	5.49E+01	1.32E+02	6.42E+01	6.09E+01	3.88E+01	1.32E+02	1.73E+02
	degree	5	7	8	2	4	6	3	1
F6	min	9.39E+02	1.19E+03	1.44E+03	9.56E+02	9.15E+02	1.13E+03	8.50E+02	9.22E+02
	mean	1.21E+03	1.32E+03	1.54E+03	1.17E+03	1.10E+03	1.24E+03	1.02E+03	**1.01E+03**
	std	1.35E+04	3.74E+03	4.14E+03	7.75E+03	4.44E+03	3.25E+03	9.42E+03	6.08E+03
	degree	5	7	8	4	3	6	2	1
F7	min	9.14E+02	9.37E+02	1.16E+03	9.35E+02	8.88E+02	1.01E+03	9.06E+02	9.02E+02
	mean	9.84E+02	9.80E+02	1.22E+03	9.78E+02	9.40E+02	1.09E+03	1.03E+03	**9.38E+02**
	std	8.78E+02	6.66E+02	1.76E+03	7.88E+02	5.80E+02	6.34E+02	3.92E+03	7.66E+02
	degree	5	4	8	3	2	7	6	1
F8	min	4.61E+03	7.06E+03	1.19E+04	1.95E+03	2.41E+03	5.13E+03	2.78E+03	2.85E+03
	mean	5.35E+03	8.94E+03	1.70E+04	3.79E+03	4.10E+03	8.80E+03	6.33E+03	**3.59E+03**
	std	3.65E+04	1.81E+06	7.24E+06	1.84E+06	5.89E+05	3.03E+06	3.88E+06	2.78E+06
	degree	4	7	8	2	3	6	5	1
F9	min	3.84E+03	4.09E+03	8.81E+03	4.99E+03	3.60E+03	8.08E+03	3.90E+03	3.46E+03
	mean	5.28E+03	6.21E+03	1.03E+04	8.13E+03	**4.75E+03**	8.86E+03	6.43E+03	5.16E+03
	std	3.65E+05	1.06E+06	3.63E+05	1.02E+06	3.25E+05	1.74E+05	1.60E+06	5.93E+05
	degree	3	4	8	6	1	7	5	2
F10	min	1.17E+03	1.32E+03	8.50E+03	1.25E+03	1.15E+03	2.37E+03	1.32E+03	1.17E+03
	mean	1.31E+03	1.59E+03	2.60E+04	1.43E+03	1.30E+03	3.97E+03	2.01E+03	**1.29E+03**
	std	4.27E+03	2.98E+04	1.23E+08	3.44E+04	1.86E+04	1.76E+06	7.70E+05	8.34E+03
	degree	3	5	8	4	2	7	6	1
F11	min	4.65E+04	6.15E+06	9.92E+09	8.46E+05	2.17E+05	1.14E+09	4.15E+05	1.61E+05
	mean	**9.95E+05**	1.12E+08	2.10E+10	1.21E+07	2.20E+06	2.52E+09	1.36E+08	2.10E+06
	std	7.92E+11	7.91E+15	3.65E+19	2.05E+14	3.93E+12	9.86E+17	4.44E+16	2.82E+12
	degree	1	5	8	4	3	7	6	2
F12	min	4.17E+03	5.08E+05	1.04E+10	5.62E+03	3.66E+03	3.09E+08	5.13E+04	4.87E+03
	mean	2.88E+04	1.77E+06	2.15E+10	4.19E+04	**1.40E+04**	1.10E+09	7.25E+06	4.35E+04
	std	5.55E+08	1.17E+13	4.89E+19	2.58E+09	1.82E+08	1.29E+17	2.01E+14	3.19E+09
	degree	2	5	8	3	1	7	6	4
F13	min	3.89E+03	1.71E+04	1.51E+06	2.01E+03	1.63E+03	1.34E+05	1.40E+04	7.95E+03
	mean	6.21E+04	1.04E+06	3.07E+07	2.23E+04	**7.88E+03**	6.74E+05	2.65E+05	1.19E+05
	std	3.16E+09	1.39E+12	4.67E+14	9.47E+08	2.32E+08	1.90E+11	1.23E+11	9.63E+09
	degree	3	7	8	2	1	6	5	4
F14	min	2.03E+03	5.04E+04	7.91E+08	2.52E+03	1.67E+03	4.13E+06	6.90E+03	2.43E+03
	mean	1.58E+04	1.25E+05	3.27E+09	1.10E+04	6.06E+03	5.84E+07	8.75E+04	**5.91E+03**
	std	1.78E+08	3.32E+09	2.47E+18	5.30E+07	4.08E+07	2.83E+15	5.77E+09	6.79E+08
	degree	4	6	8	3	2	7	5	1
F15	min	2.34E+03	2.58E+03	4.62E+03	2.77E+03	1.92E+03	3.38E+03	2.83E+03	2.16E+03
	mean	2.91E+03	3.56E+03	5.70E+03	3.42E+03	2.90E+03	4.09E+03	3.45E+03	**2.90E+03**
	std	1.19E+05	2.91E+05	4.16E+05	6.94E+04	1.18E+05	7.99E+04	7.50E+04	1.79E+05
	degree	3	6	8	4	2	7	5	1
F16	min	2.04E+03	1.93E+03	3.19E+03	1.86E+03	1.92E+03	2.35E+03	2.01E+03	1.96E+03
	mean	2.48E+03	2.77E+03	4.18E+03	2.22E+03	2.38E+03	2.82E+03	2.67E+03	**2.17E+03**
	std	5.65E+04	7.72E+04	7.24E+05	3.22E+04	6.80E+04	4.30E+04	9.22E+04	8.72E+04
	degree	4	6	8	2	3	7	5	1
F17	min	9.25E+04	1.09E+05	3.25E+07	7.86E+04	2.34E+04	2.71E+06	7.52E+04	7.55E+04
	mean	4.15E+05	3.56E+06	3.39E+08	3.90E+05	**1.07E+05**	1.54E+07	3.83E+06	2.09E+06
	std	1.05E+11	1.21E+13	3.90E+16	1.31E+11	1.09E+10	8.51E+13	3.14E+13	6.06E+12
	degree	3	5	8	2	1	7	6	4
F18	min	2.08E+03	2.56E+05	1.08E+09	2.25E+03	2.09E+03	1.81E+07	2.88E+03	2.48E+03
	mean	8.70E+03	1.77E+06	3.81E+09	8.82E+03	6.40E+03	9.15E+07	3.80E+06	**6.28E+03**
	std	9.27E+07	1.58E+12	2.59E+18	4.15E+07	2.07E+07	4.92E+15	1.32E+14	1.66E+08
	degree	3	5	8	4	2	7	6	1
F19	min	2.21E+03	2.25E+03	3.06E+03	2.34E+03	2.13E+03	2.62E+03	2.31E+03	2.25E+03
	mean	2.80E+03	2.85E+03	3.59E+03	2.67E+03	2.51E+03	2.94E+03	2.75E+03	**2.49E+03**
	std	9.98E+04	6.65E+04	4.99E+04	1.90E+04	3.06E+04	2.39E+04	3.76E+04	4.86E+04
	degree	5	6	8	3	2	7	4	1
F20	min	2.40E+03	2.52E+03	2.64E+03	2.41E+03	2.41E+03	2.53E+03	2.47E+03	2.41E+03
	mean	2.51E+03	2.59E+03	2.77E+03	2.47E+03	2.47E+03	2.60E+03	2.56E+03	**2.46E+03**
	std	4.95E+03	1.68E+03	4.66E+03	1.52E+03	1.07E+03	5.88E+02	2.83E+03	2.16E+03
	degree	4	6	8	2	3	7	5	1
F21	min	2.30E+03	2.59E+03	7.56E+03	2.43E+03	2.33E+03	4.09E+03	2.31E+03	2.30E+03
	mean	5.57E+03	7.30E+03	1.11E+04	2.94E+03	2.89E+03	8.97E+03	5.20E+03	**4.81E+03**
	std	5.10E+06	1.33E+06	1.63E+06	1.58E+05	1.22E+06	4.69E+06	5.96E+06	5.33E+06
	degree	5	6	8	3	2	7	4	1
F22	min	2.81E+03	3.10E+03	3.17E+03	2.81E+03	2.79E+03	3.01E+03	2.82E+03	2.78E+03
	mean	2.91E+03	3.30E+03	3.38E+03	2.90E+03	2.95E+03	3.09E+03	3.01E+03	**2.89E+03**
	std	4.94E+03	1.47E+04	1.72E+04	2.35E+03	6.17E+03	2.10E+03	4.97E+03	9.52E+03
	degree	3	7	8	2	4	6	5	1
F23	min	2.95E+03	3.25E+03	3.36E+03	3.02E+03	3.00E+03	3.18E+03	3.02E+03	2.91E+03
	mean	3.07E+03	3.48E+03	3.54E+03	3.11E+03	3.09E+03	3.26E+03	3.18E+03	**3.04E+03**
	std	6.53E+03	1.15E+04	1.70E+04	2.38E+03	4.66E+03	1.94E+03	1.01E+04	1.08E+04
	degree	2	7	8	4	3	6	5	1
F24	min	2.88E+03	2.93E+03	4.12E+03	2.99E+03	2.89E+03	3.30E+03	2.92E+03	2.88E+03
	mean	2.90E+03	3.01E+03	5.62E+03	3.09E+03	2.96E+03	3.68E+03	3.01E+03	**2.89E+03**
	std	1.98E+02	1.10E+03	7.63E+05	5.09E+03	1.89E+03	1.05E+05	3.77E+04	2.39E+02
	degree	2	4	8	6	3	7	5	1
F25	min	2.80E+03	5.54E+03	9.08E+03	5.61E+03	3.21E+03	7.08E+03	4.14E+03	2.81E+03
	mean	6.10E+03	8.28E+03	1.17E+04	6.40E+03	6.80E+03	7.93E+03	6.59E+03	**5.99E+03**
	std	2.39E+06	7.23E+05	1.34E+06	3.89E+05	1.43E+06	2.89E+05	9.41E+05	1.64E+06
	degree	2	7	8	3	5	6	4	1
F26	min	3.21E+03	3.32E+03	3.42E+03	3.24E+03	3.26E+03	3.45E+03	3.24E+03	3.21E+03
	mean	3.27E+03	3.64E+03	4.08E+03	3.32E+03	3.36E+03	3.57E+03	3.38E+03	**3.25E+03**
	std	2.22E+03	8.23E+04	7.92E+04	1.49E+03	1.04E+04	3.19E+03	1.11E+04	8.21E+03
	degree	2	7	8	3	4	6	5	1
F27	min	3.20E+03	3.36E+03	5.37E+03	3.38E+03	3.27E+03	3.83E+03	3.31E+03	3.22E+03
	mean	**3.23E+03**	3.48E+03	7.46E+03	3.54E+03	3.34E+03	4.50E+03	3.74E+03	3.26E+03
	std	6.25E+02	7.30E+03	1.25E+06	2.66E+04	3.29E+03	2.15E+05	8.11E+05	8.90E+02
	degree	1	4	8	5	3	7	6	2
F28	min	3.74E+03	4.39E+03	5.31E+03	3.84E+03	3.50E+03	4.65E+03	3.83E+03	3.66E+03
	mean	**4.18E+03**	4.93E+03	8.02E+03	4.20E+03	4.28E+03	5.20E+03	4.58E+03	4.29E+03
	std	6.17E+04	1.49E+05	3.79E+06	3.14E+04	1.27E+05	7.25E+04	1.80E+05	8.12E+04
	degree	1	6	8	2	3	7	5	4
F29	min	6.19E+03	3.69E+05	5.43E+08	2.34E+04	7.12E+03	8.89E+07	1.68E+04	6.38E+03
	mean	1.60E+04	1.24E+07	2.54E+09	2.19E+05	3.10E+04	2.04E+08	3.02E+06	**1.59E+04**
	std	3.36E+07	1.93E+14	1.69E+18	3.51E+10	6.25E+08	6.53E+15	2.08E+13	8.59E+10
	degree	2	6	8	4	3	7	5	1

**Table 4:** CEC2017 test results: 100 dimensions.

		Dim = 30							
		SSA	HHO	BOA	OMA	WOA	SCA	DBO	SCDBO
F1	min	2.04E+08	3.72E+10	2.88E+11	7.48E+10	4.16E+10	1.87E+11	2.40E+10	4.41E+09
	mean	**3.74E+08**	4.81E+10	2.95E+11	1.13E+11	6.41E+10	2.13E+11	8.14E+10	1.43E+10
	std	1.76E+16	3.51E+19	1.23E+19	3.76E+20	1.47E+20	2.15E+20	4.44E+21	3.05E+19
	degree	1	3	8	6	4	7	5	2
F2	min	3.58E+05	3.10E+05	9.91E+05	3.67E+05	1.82E+05	4.38E+05	3.65E+05	3.19E+05
	mean	7.37E+05	3.66E+05	3.59E+09	4.39E+05	**2.42E+05**	6.14E+05	6.55E+05	3.50E+05
	std	2.35E+10	2.31E+10	6.83E+19	1.33E+09	4.39E+08	8.56E+09	6.71E+10	1.35E+10
	degree	7	3	8	4	1	5	6	2
F3	min	8.54E+02	5.44E+03	7.03E+04	9.71E+03	2.69E+03	3.72E+04	3.60E+03	1.39E+03
	mean	2.42E+03	9.70E+03	1.18E+05	1.83E+04	5.58E+03	5.37E+04	2.10E+04	**2.13E+03**
	std	7.08E+03	4.62E+06	5.31E+08	1.61E+07	3.85E+06	7.79E+07	2.37E+08	2.05E+05
	degree	2	4	8	5	3	7	6	1
F4	min	1.29E+03	1.57E+03	2.09E+03	1.44E+03	1.35E+03	1.94E+03	1.37E+03	1.27E+03
	mean	1.44E+03	1.69E+03	2.29E+03	1.67E+03	1.46E+03	2.04E+03	1.68E+03	**1.42E+03**
	std	2.02E+03	2.69E+03	7.00E+03	1.35E+04	3.61E+03	3.20E+03	4.68E+04	7.58E+03
	degree	2	6	8	4	3	7	5	1
F5	min	6.62E+02	6.85E+02	7.16E+02	6.67E+02	6.61E+02	6.92E+02	6.61E+02	6.55E+02
	mean	6.66E+02	6.90E+02	7.28E+02	6.84E+02	6.70E+02	7.06E+02	6.79E+02	**6.65E+02**
	std	3.64E+00	1.09E+01	2.81E+01	6.24E+01	1.44E+01	3.43E+01	1.05E+02	2.61E+01
	degree	2	6	8	5	3	7	4	1
F6	min	2.99E+03	3.39E+03	4.13E+03	3.33E+03	2.81E+03	3.73E+03	2.48E+03	2.61E+03
	mean	3.24E+03	3.79E+03	4.25E+03	4.13E+03	3.21E+03	4.18E+03	2.93E+03	**2.79E+03**
	std	1.11E+04	2.05E+04	2.71E+03	2.25E+05	1.54E+04	6.03E+04	2.35E+04	2.90E+04
	degree	4	5	8	6	3	7	2	1
F7	min	1.75E+03	1.93E+03	2.59E+03	1.88E+03	1.67E+03	2.29E+03	1.76E+03	1.70E+03
	mean	1.85E+03	2.13E+03	2.78E+03	2.01E+03	1.87E+03	2.42E+03	2.22E+03	**1.84E+03**
	std	2.43E+03	4.37E+03	7.69E+03	1.12E+04	5.62E+03	3.34E+03	4.54E+04	6.50E+03
	degree	2	5	8	4	3	7	6	1
F8	min	2.43E+04	5.69E+04	9.47E+04	5.50E+04	2.32E+04	7.67E+04	5.05E+04	3.76E+04
	mean	**2.54E+04**	6.88E+04	1.09E+05	7.06E+04	3.03E+04	9.12E+04	7.51E+04	6.58E+04
	std	4.08E+05	2.71E+07	7.68E+07	5.98E+07	1.31E+07	7.41E+07	9.35E+07	2.23E+08
	degree	1	4	8	5	2	7	6	3
F9	min	1.39E+04	2.11E+04	3.35E+04	3.06E+04	1.70E+04	3.20E+04	1.85E+04	1.76E+04
	mean	**1.72E+04**	2.41E+04	3.55E+04	3.23E+04	1.93E+04	3.31E+04	2.93E+04	2.29E+04
	std	2.61E+06	2.83E+06	1.03E+06	4.74E+05	1.57E+06	3.33E+05	2.20E+07	2.69E+07
	degree	1	4	8	6	2	7	5	3
F10	min	3.49E+04	7.01E+04	3.83E+05	6.11E+04	1.75E+04	1.42E+05	1.23E+05	9.83E+04
	mean	7.50E+04	1.48E+05	1.26E+07	9.67E+04	**3.48E+04**	1.91E+05	2.10E+05	1.96E+05
	std	3.73E+08	1.84E+09	9.39E+14	3.31E+08	1.46E+08	1.62E+09	2.14E+09	2.28E+09
	degree	2	4	8	3	1	5	7	6
F11	min	4.80E+07	5.26E+09	2.00E+11	1.35E+10	8.70E+08	7.69E+10	3.54E+09	2.85E+08
	mean	4.69E+08	1.17E+10	2.45E+11	2.32E+10	6.96E+09	9.66E+10	6.96E+09	**3.91E+08**
	std	4.64E+15	1.17E+19	2.46E+20	7.11E+19	3.35E+19	1.25E+20	4.81E+18	3.99E+16
	degree	2	5	8	6	4	7	3	1
F12	min	2.35E+04	6.50E+07	3.49E+10	1.85E+08	3.41E+05	1.24E+10	1.61E+07	8.29E+04
	mean	1.93E+05	2.53E+08	6.14E+10	9.53E+08	6.34E+08	1.83E+10	2.74E+08	**1.84E+05**
	std	1.20E+10	3.73E+16	5.91E+19	4.36E+17	1.88E+18	9.14E+18	4.06E+16	3.78E+10
	degree	2	3	8	6	5	7	4	1
F13	min	7.63E+05	4.06E+06	7.84E+07	1.53E+06	7.16E+05	2.90E+07	3.06E+06	6.35E+05
	mean	2.15E+06	1.06E+07	3.91E+08	4.09E+06	**1.72E+06**	7.37E+07	1.41E+07	3.97E+06
	std	1.05E+12	1.20E+13	3.52E+16	3.92E+12	7.32E+11	1.02E+15	7.51E+13	5.93E+12
	degree	2	5	8	4	1	7	6	3
F14	min	7.73E+03	6.25E+06	2.43E+10	3.75E+06	1.99E+04	3.48E+09	6.65E+04	2.15E+04
	mean	2.44E+04	3.38E+07	3.67E+10	6.24E+07	7.21E+05	6.11E+09	6.16E+07	**2.39E+04**
	std	3.41E+08	1.07E+16	2.02E+19	1.47E+16	1.75E+12	2.18E+18	6.98E+15	9.89E+08
	degree	2	4	8	6	3	7	5	1
F15	min	5.13E+03	7.78E+03	1.67E+04	7.91E+03	5.95E+03	1.34E+04	6.94E+03	5.16E+03
	mean	6.57E+03	9.96E+03	2.49E+04	1.00E+04	7.42E+03	1.48E+04	9.52E+03	**6.42E+03**
	std	5.50E+05	1.60E+06	1.48E+07	1.76E+06	9.45E+05	1.27E+06	2.39E+06	8.80E+05
	degree	2	5	8	6	3	7	4	1
F16	min	5.08E+03	6.31E+03	2.52E+06	5.14E+03	5.11E+03	1.67E+04	5.78E+03	4.54E+03
	mean	6.04E+03	8.43E+03	3.48E+07	6.67E+03	7.27E+03	7.96E+04	9.05E+03	**6.02E+03**
	std	3.98E+05	3.14E+06	8.66E+14	1.56E+06	2.31E+06	7.90E+09	2.39E+06	5.08E+05
	degree	2	5	8	3	4	7	6	1
F17	min	6.34E+05	2.33E+06	2.06E+08	1.55E+06	9.08E+05	4.74E+07	4.16E+06	2.56E+06
	mean	2.92E+06	1.17E+07	7.54E+08	4.90E+06	**2.72E+06**	1.19E+08	2.19E+07	7.13E+06
	std	1.34E+12	4.39E+13	9.79E+16	3.99E+12	1.36E+12	1.55E+15	1.79E+14	2.00E+13
	degree	2	5	8	3	1	7	6	4
F18	min	2.93E+03	1.15E+07	2.44E+10	9.07E+06	5.42E+04	3.14E+09	3.58E+06	2.95E+03
	mean	1.83E+05	4.26E+07	3.53E+10	6.80E+07	7.97E+06	5.76E+09	8.99E+07	**3.94E+04**
	std	7.71E+08	5.99E+14	3.88E+19	2.41E+15	7.02E+14	2.45E+18	7.52E+15	3.59E+12
	degree	2	4	8	5	3	7	6	1
F19	min	5.02E+03	4.91E+03	8.03E+03	6.78E+03	4.16E+03	7.55E+03	5.56E+03	5.21E+03
	mean	6.00E+03	6.09E+03	9.19E+03	7.53E+03	**5.21E+03**	8.12E+03	7.44E+03	6.37E+03
	std	3.17E+05	2.80E+05	1.96E+05	6.57E+04	2.62E+05	8.46E+04	5.16E+05	3.35E+05
	degree	2	3	8	6	1	7	5	4
F20	min	3.27E+03	3.77E+03	4.56E+03	3.26E+03	3.37E+03	4.03E+03	3.60E+03	3.12E+03
	mean	3.61E+03	4.37E+03	5.01E+03	3.48E+03	3.61E+03	4.20E+03	4.03E+03	**3.42E+03**
	std	4.52E+04	5.33E+04	5.97E+04	9.57E+03	1.92E+04	9.90E+03	4.90E+04	2.94E+04
	degree	3	7	8	2	4	6	5	1
F21	min	1.59E+04	2.40E+04	3.63E+04	3.31E+04	2.13E+04	3.46E+04	2.19E+04	1.96E+04
	mean	2.37E+04	2.74E+04	3.80E+04	3.48E+04	2.40E+04	3.56E+04	2.97E+04	**2.32E+04**
	std	2.68E+06	4.18E+06	7.04E+05	3.53E+05	1.59E+06	2.52E+05	1.87E+07	2.19E+07
	degree	2	4	8	6	3	7	5	1
F22	min	3.86E+03	4.87E+03	5.30E+03	3.91E+03	4.22E+03	4.96E+03	4.37E+03	3.78E+03
	mean	4.18E+03	5.78E+03	6.40E+03	4.15E+03	4.63E+03	5.24E+03	4.83E+03	**4.14E+03**
	std	3.79E+04	1.91E+05	4.83E+05	1.79E+04	5.47E+04	2.79E+04	6.89E+04	1.09E+05
	degree	3	7	8	2	4	6	5	1
F23	min	4.49E+03	7.00E+03	7.24E+03	5.13E+03	5.23E+03	6.89E+03	5.40E+03	4.68E+03
	mean	5.77E+03	8.25E+03	9.81E+03	5.81E+03	5.99E+03	7.41E+03	5.93E+03	**5.67E+03**
	std	1.31E+05	5.40E+05	4.72E+06	1.39E+05	3.12E+05	1.04E+05	1.34E+05	4.70E+05
	degree	2	7	8	3	5	6	4	1
F24	min	3.58E+03	5.69E+03	2.61E+04	9.09E+03	5.11E+03	1.72E+04	5.42E+03	4.28E+03
	mean	4.88E+03	6.87E+03	3.24E+04	1.17E+04	7.96E+03	2.22E+04	8.59E+03	**4.86E+03**
	std	6.57E+03	2.84E+05	5.31E+06	2.02E+06	2.46E+06	1.11E+07	2.31E+07	1.17E+05
	degree	2	3	8	6	4	7	5	1
F25	min	5.16E+03	2.57E+04	5.42E+04	2.67E+04	2.86E+04	3.51E+04	2.16E+04	1.59E+04
	mean	2.19E+04	3.16E+04	5.98E+04	3.73E+04	3.19E+04	4.17E+04	2.74E+04	**2.13E+04**
	std	3.63E+07	5.94E+06	1.04E+07	3.06E+07	4.48E+06	8.41E+06	1.61E+07	9.88E+06
	degree	2	4	8	6	5	7	3	1
F26	min	3.54E+03	4.84E+03	8.47E+03	4.84E+03	4.45E+03	7.69E+03	3.79E+03	3.56E+03
	mean	3.79E+03	7.14E+03	1.17E+04	5.44E+03	5.33E+03	8.80E+03	4.65E+03	**3.71E+03**
	std	2.68E+04	2.59E+06	4.53E+06	1.02E+05	3.49E+05	2.84E+05	1.87E+05	2.38E+05
	degree	2	6	8	5	4	7	3	1
F27	min	3.67E+03	7.79E+03	3.38E+04	1.15E+04	6.37E+03	2.29E+04	7.15E+03	4.57E+03
	mean	**3.80E+03**	9.40E+03	3.78E+04	1.50E+04	9.30E+03	2.74E+04	1.74E+04	5.75E+03
	std	8.45E+03	7.91E+05	7.79E+06	3.84E+06	2.18E+06	7.08E+06	3.98E+07	6.13E+05
	degree	1	4	8	5	3	7	6	2
F28	min	6.39E+03	9.84E+03	9.33E+04	1.05E+04	8.61E+03	2.08E+04	7.83E+03	6.74E+03
	mean	7.63E+03	1.31E+04	1.63E+06	1.32E+04	1.07E+04	3.56E+04	1.16E+04	**7.59E+03**
	std	4.87E+05	2.85E+06	2.46E+12	2.05E+06	1.53E+06	3.29E+08	7.10E+06	2.82E+05
	degree	2	5	8	6	3	7	4	1
F29	min	1.49E+05	2.41E+08	4.12E+10	1.63E+08	5.02E+06	9.22E+09	4.56E+07	6.12E+05
	mean	**9.94E+05**	6.72E+08	5.64E+10	1.42E+09	1.78E+08	1.37E+10	3.08E+08	2.30E+06
	std	5.34E+11	1.02E+17	3.17E+19	2.20E+18	1.22E+17	6.08E+18	2.72E+16	1.73E+12
	degree	1	5	8	6	3	7	4	2

**Table 5:** Wilcoxon rank sum test (Dim = 30).

	SSA	HHO	BOA	OMA	WOA	SCA	DBO
F1	3.47E-10 < 0.05	3.02E-11 < 0.05	3.00E-11 < 0.05	3.02E-11 < 0.05	3.02E-11 < 0.05	3.02E-11 < 0.05	4.98E-11 < 0.05
F2	2.92E-09 < 0.05	2.68E-06 < 0.05	4.50E-11 < 0.05	6.52E-01	3.02E-11 < 0.05	1.17E-03 < 0.05	1.07E-07 < 0.05
F3	6.00E-01	8.99E-11 < 0.05	3.02E-11 < 0.05	3.34E-11 < 0.05	7.04E-07 < 0.05	3.02E-11 < 0.05	3.20E-09 < 0.05
F4	2.43E-05 < 0.05	7.39E-11 < 0.05	3.02E-11 < 0.05	8.68E-03 < 0.05	1.09E-01 < 0.05	3.02E-11 < 0.05	6.53E-07 < 0.05
F5	1.56E-02 < 0.05	2.37E-10 < 0.05	3.02E-11 < 0.05	4.43E-03 < 0.05	7.96E-03 < 0.05	1.10E-08 < 0.05	5.83E-03 < 0.05
F6	2.68E-06 < 0.05	8.99E-11 < 0.05	3.02E-11 < 0.05	8.35E-08 < 0.05	3.18E-03 < 0.05	5.07E-10 < 0.05	7.96E-01
F7	3.83E-05 < 0.05	1.73E-07 < 0.05	3.02E-11 < 0.05	1.68E-04 < 0.05	6.52E-01 < 0.05	3.02E-11 < 0.05	9.51E-06 < 0.05
F8	9.47E-01	7.69E-08 < 0.05	3.02E-11 < 0.05	3.77E-04 < 0.05	8.12E-04 < 0.05	1.49E-04 < 0.05	1.91E-01
F9	4.46E-01	8.12E-04 < 0.05	3.02E-11 < 0.05	8.89E-10 < 0.05	9.51E-06 < 0.05	3.02E-11 < 0.05	6.91E-04 < 0.05
F10	2.77E-01	1.87E-07 < 0.05	3.02E-11 < 0.05	1.50E-02 < 0.05	8.31E-03 < 0.05	3.02E-11 < 0.05	2.15E-10 < 0.05
F11	7.96E-03 < 0.05	3.02E-11 < 0.05	3.02E-11 < 0.05	5.97E-09 < 0.05	5.01E-01	3.02E-11 < 0.05	4.50E-11 < 0.05
F12	3.16E-05 < 0.05	3.02E-11 < 0.05	3.02E-11 < 0.05	4.04E-01	5.09E-06 < 0.05	3.02E-11 < 0.05	1.69E-09 < 0.05
F13	3.85E-03 < 0.05	5.46E-06 < 0.05	3.02E-11 < 0.05	3.26E-07 < 0.05	3.82E-10 < 0.05	3.50E-09 < 0.05	5.94E-02
F14	3.39E-02 < 0.05	4.20E-10 < 0.05	3.02E-11 < 0.05	6.95E-01	5.57E-03 < 0.05	3.02E-11 < 0.05	2.00E-06 < 0.05
F15	7.84E-01	4.12E-06 < 0.05	3.69E-11 < 0.05	6.67E-03 < 0.05	2.61E-02 < 0.05	1.78E-10 < 0.05	5.75E-02
F16	5.11E-01	2.89E-03 < 0.05	4.98E-11 < 0.05	2.25E-04 < 0.05	2.84E-01	7.22E-06 < 0.05	5.40E-01
F17	4.03E-03 < 0.05	1.53E-05 < 0.05	3.02E-11 < 0.05	5.97E-05 < 0.05	4.57E-09 < 0.05	4.50E-11 < 0.05	1.63E-02
F18	8.07E-01	3.02E-11 < 0.05	3.02E-11 < 0.05	7.96E-01	6.95E-01	3.02E-11 < 0.05	3.82E-09 < 0.05
F19	9.47E-01	8.30E-01	8.99E-11 < 0.05	3.63E-01	1.52E-03 < 0.05	1.22E-02 < 0.05	1.00E-04 < 0.05
F20	1.30E-01	3.08E-08 < 0.05	3.02E-11 < 0.05	7.17E-01	3.95E-01	6.72E-10 < 0.05	1.39E-06 < 0.05
F21	9.00E-01	1.19E-06 < 0.05	3.69E-11 < 0.05	2.71E-02 < 0.05	3.51E-02 < 0.05	7.77E-09 < 0.05	7.62E-01
F22	9.47E-01	7.39E-11 < 0.05	3.69E-11 < 0.05	1.62E-01	1.67E-01	1.29E-09 < 0.05	3.57E-06 < 0.05
F23	1.30E-03 < 0.05	3.02E-11 < 0.05	3.02E-11 < 0.05	9.05E-02	2.28E-01	2.87E-10 < 0.05	1.22E-02 < 0.05
F24	6.35E-02	3.02E-11 < 0.05	3.02E-11 < 0.05	3.02E-11 < 0.05	2.00E-06 < 0.05	3.02E-11 < 0.05	2.67E-09 < 0.05
F25	1.33E-01	3.82E-09 < 0.05	3.02E-11 < 0.05	4.36E-02 < 0.05	9.88E-03 < 0.05	1.01E-08 < 0.05	2.53E-04 < 0.05
F26	4.43E-03 < 0.05	2.44E-09 < 0.05	3.02E-11 < 0.05	3.11E-01	1.99E-02 < 0.05	4.50E-11 < 0.05	2.77E-01
F27	2.77E-05 < 0.05	3.34E-11 < 0.05	3.02E-11 < 0.05	3.34E-11 < 0.05	5.00E-09 < 0.05	3.02E-11 < 0.05	6.07E-11 < 0.05
F28	5.01E-01	2.78E-07 < 0.05	3.02E-11 < 0.05	4.20E-01	5.08E-03 < 0.05	4.08E-11 < 0.05	9.88E-03 < 0.05
F29	3.78E-02 < 0.05	3.02E-11 < 0.05	3.02E-11 < 0.05	1.29E-09 < 0.05	9.93E-02 < 0.05	3.02E-11 < 0.05	7.09E-08 < 0.05

**Table 6:** Wilcoxon rank sum test (Dim = 100).

	SSA	HHO	BOA	OMA	WOA	SCA	DBO
F1	3.02E-11 < 0.05	3.02E-11 < 0.05	2.92E-11 < 0.05	3.02E-11 < 0.05	3.02E-11 < 0.05	3.02E-11 < 0.05	3.02E-11 < 0.05
F2	3.83E-06 < 0.05	1.20E-08 < 0.05	3.34E-11 < 0.05	1.67E-01	3.02E-11 < 0.05	2.50E-03 < 0.05	7.48E-02
F3	3.02E-11 < 0.05	3.02E-11 < 0.05	3.02E-11 < 0.05	3.02E-11 < 0.05	3.34E-11 < 0.05	3.02E-11 < 0.05	3.02E-11 < 0.05
F4	4.46E-04 < 0.05	3.02E-11 < 0.05	3.02E-11 < 0.05	3.69E-11 < 0.05	1.62E-01	3.02E-11 < 0.05	5.86E-06 < 0.05
F5	9.47E-01	3.02E-11 < 0.05	3.02E-11 < 0.05	8.99E-11 < 0.05	6.97E-03 < 0.05	3.02E-11 < 0.05	5.86E-06 < 0.05
F6	6.01E-08 < 0.05	3.02E-11 < 0.05	3.02E-11 < 0.05	3.02E-11 < 0.05	1.11E-04 < 0.05	3.02E-11 < 0.05	1.05E-01
F7	7.96E-01	5.49E-11 < 0.05	3.02E-11 < 0.05	2.60E-05 < 0.05	5.30E-01	3.02E-11 < 0.05	1.29E-06 < 0.05
F8	3.02E-11 < 0.05	1.71E-01	4.50E-11 < 0.05	3.39E-02 < 0.05	1.78E-10 < 0.05	5.46E-09 < 0.05	1.86E-03 < 0.05
F9	8.10E-10 < 0.05	1.58E-01	3.34E-11 < 0.05	2.67E-09 < 0.05	2.01E-04 < 0.05	3.47E-10 < 0.05	1.99E-02 < 0.05
F10	3.69E-11 < 0.05	3.96E-08 < 0.05	3.02E-11 < 0.05	4.50E-11 < 0.05	3.02E-11 < 0.05	2.13E-04 < 0.05	6.95E-01
F11	1.41E-09 < 0.05	3.02E-11 < 0.05	3.02E-11 < 0.05	3.02E-11 < 0.05	3.02E-11 < 0.05	3.02E-11 < 0.05	3.02E-11 < 0.05
F12	1.07E-09 < 0.05	3.02E-11 < 0.05	3.01E-11 < 0.05	3.02E-11 < 0.05	3.34E-11 < 0.05	3.02E-11 < 0.05	3.02E-11 < 0.05
F13	3.83E-06 < 0.05	1.09E-10 < 0.05	3.02E-11 < 0.05	1.26E-01	2.43E-05 < 0.05	3.02E-11 < 0.05	7.12E-09 < 0.05
F14	1.75E-05 < 0.05	3.02E-11 < 0.05	3.01E-11 < 0.05	3.02E-11 < 0.05	2.25E-04 < 0.05	3.02E-11 < 0.05	3.02E-11 < 0.05
F15	2.01E-01	3.69E-11 < 0.05	3.02E-11 < 0.05	9.76E-10 < 0.05	2.49E-06 < 0.05	3.02E-11 < 0.05	4.62E-10 < 0.05
F16	2.34E-01	1.85E-08 < 0.05	3.02E-11 < 0.05	9.35E-01	9.33E-02	3.02E-11 < 0.05	1.96E-10 < 0.05
F17	4.11E-07 < 0.05	9.03E-04 < 0.05	3.02E-11 < 0.05	1.33E-01	3.01E-07 < 0.05	3.02E-11 < 0.05	4.57E-09 < 0.05
F18	4.42E-06 < 0.05	3.02E-11 < 0.05	3.01E-11 < 0.05	3.02E-11 < 0.05	9.06E-08 < 0.05	3.02E-11 < 0.05	3.02E-11 < 0.05
F19	4.68E-02 < 0.05	2.62E-03 < 0.05	3.34E-11 < 0.05	2.88E-06 < 0.05	2.83E-08 < 0.05	5.07E-10 < 0.05	2.00E-05 < 0.05
F20	6.55E-04 < 0.05	3.34E-11 < 0.05	3.02E-11 < 0.05	9.59E-01	3.92E-02 < 0.05	4.08E-11 < 0.05	2.37E-10 < 0.05
F21	1.29E-06 < 0.05	6.55E-04 < 0.05	3.02E-11 < 0.05	3.69E-11 < 0.05	1.41E-01	3.02E-11 < 0.05	1.43E-05 < 0.05
F22	3.34E-03 < 0.05	3.02E-11 < 0.05	3.02E-11 < 0.05	1.52E-03 < 0.05	1.02E-01	4.98E-11 < 0.05	2.60E-05 < 0.05
F23	8.68E-03 < 0.05	3.02E-11 < 0.05	3.02E-11 < 0.05	2.81E-02 < 0.05	8.56E-04 < 0.05	9.92E-11 < 0.05	1.89E-04 < 0.05
F24	3.02E-11 < 0.05	3.34E-11 < 0.05	3.02E-11 < 0.05	3.02E-11 < 0.05	3.34E-11 < 0.05	3.02E-11 < 0.05	2.87E-10 < 0.05
F25	5.49E-01	3.34E-11 < 0.05	3.02E-11 < 0.05	3.69E-11 < 0.05	3.69E-11 < 0.05	3.02E-11 < 0.05	6.15E-02
F26	5.89E-01	3.02E-11 < 0.05	3.02E-11 < 0.05	4.08E-11 < 0.05	1.21E-10 < 0.05	3.02E-11 < 0.05	6.52E-09 < 0.05
F27	3.02E-11 < 0.05	3.02E-11 < 0.05	3.02E-11 < 0.05	3.02E-11 < 0.05	3.02E-11 < 0.05	3.02E-11 < 0.05	4.08E-11 < 0.05
F28	8.07E-01	3.02E-11 < 0.05	3.02E-11 < 0.05	3.02E-11 < 0.05	3.02E-11 < 0.05	3.02E-11 < 0.05	3.02E-11 < 0.05
F29	1.87E-07 < 0.05	3.02E-11 < 0.05	3.01E-11 < 0.05	3.02E-11 < 0.05	6.70E-11 < 0.05	3.02E-11 < 0.05	3.02E-11 < 0.05

**Table 7:** Robotic arm parameters.

Algorithms	Optimum Variables							Force Difference	Ranking
	$x_{1}\left(a\right)$	$x_{2}\left(b\right)$	$x_{3}\left(c\right)$	$x_{4}\left(e\right)$	$x_{5}\left(f\right)$	$x_{6}\left(l\right)$	$x_{7}\left(\delta \right)$		
SSA	1.50E+02	1.31E+02	1.00E+02	1.92E+01	3.38E+01	1.00E+02	1.97E+00	5.35E+00	7
HHO	9.95E+01	3.77E+01	1.01E+02	0.00E+00	1.00E+01	1.00E+02	1.20E+00	1.51E-16	4
BOA	1.50E+02	1.50E+02	1.00E+02	0.00E+00	1.50E+02	1.00E+02	3.14E+00	8.58E+00	8
OMA	1.49E+02	1.42E+02	2.09E+02	6.35E+00	1.76E+02	1.29E+02	2.66E+00	3.33E+00	6
WOA	1.00E+02	3.82E+01	1.00E+02	0.00E+00	1.03E+01	1.00E+02	1.08E+00	1.45E-16	3
SCA	9.10E+01	2.56E+01	1.60E+02	0.00E+00	1.81E+01	1.00E+02	1.75E+00	2.54E-16	5
DBO	9.37E+01	3.19E+01	2.00E+02	0.00E+00	1.00E+01	1.00E+02	1.70E+00	1.19E-16	2
SCDBO	1.00E+02	3.80E+01	1.00E+02	0.00E+00	8.40E+01	1.00+E02	2.00E+00	1.07E-16	1

**Table 8:** Statistical measurement analysis of robotic arm clamping force.

	SSA	HHO	BOA	OMA	WOA	SCA	DBO	SCDBO
Mean	2.90E+00	1.11E+01	2.64E+104	3.67E+00	9.20E-02	2.29E-16	1.81E-16	**1.31E-16**
Std	4.12E+00	3.99E+02	2.19E+209	5.02E-01	2.54E-01	7.14E-33	1.80E-32	7.93E-32
Min	7.27E-17	1.61E-16	8.58E+00	4.78E-01	7.27E-17	9.03E-17	7.27E-17	7.18E-17
Max	6.67E+00	7.91E+01	2.19E+105	4.53E+00	2.76E+00	4.97E-16	5.43E-16	1.08E-15

**Table 9:** Statistical analysis of pressure vessel variables.

Algorithms	Optimum Variables				Best Value	Ranking
	$x_{1}$	$x_{2}$	$x_{3}$	$x_{4}$		
SSA	1	0	51	89	6.35E+03	5
HHO	1	1	55	63	6.81E+03	7
BOA	2	19	48	141	8.93E+04	8
OMA	1	0	40	198	5.91E+03	3
WOA	1	0	40	200	5.89E+03	2
SCA	1	0	46	141	6.50E+03	6
DBO	1	0	44	160	6.00E+03	4
SCDBO	1	0	41	196	5.89E+03	1

**Table 10:** Statistical analysis of pressure vessel across 100 repetitions.

	SSA	HHO	BOA	OMA	WOA	SCA	DBO	SCDBO
Mean	6.35E+03	6.88E+03	5.98E+05	5.99E+03	5.93E+03	7.60E+03	6.36E+03	**5.91E+03**
Std	2.49E+05	1.64E+05	2.26E+11	5.71E+03	1.83E+02	7.15E+05	4.15E+05	2.94E+05
Min	5.90E+03	6.12E+03	6.71E+04	5.90E+03	5.97E+03	6.32E+03	5.89E+03	5.89E+03
Max	7.32E+03	7.68E+03	2.33E+06	6.17E+03	5.89E+03	9.01E+03	7.32E+03	7.32E+03

**Table 11:** Statistical table of 100 independent repetitions in UAV path planning.

		SSA	HHO	BOA	OMA	WOA	SCA	DBO	SCDBO
Scene1	Mean	1.07E+04	4.20E+11	5.10E+11	2.70E+11	8.52E+03	1.20E+11	1.16E+04	**8.13E+03**
	Std	1.61E+06	2.09E+23	2.06E+23	1.76E+23	2.25E+05	9.68E+22	2.47E+06	1.28E+04
	Min	8.88E+03	7.93E+03	1.43E+04	8.25E+03	7.89E+03	1.02E+04	9.30E+03	8.01E+03
	Ranking	3	7	8	6	2	5	4	1
Scene2	Mean	3.30E+11	7.20E+11	1.05E+12	7.80E+11	1.37E+04	8.40E+11	1.39E+04	**9.81E+03**
	Std	3.06E+23	1.34E+23	1.72E+23	9.68E+22	3.98E+06	5.21E+22	1.37E+06	2.70E+22
	Min	1.10E+04	1.29E+04	1.83E+04	1.26E+04	1.00E+04	1.28E+04	1.22E+04	9.56E+03
	Ranking	4	5	8	6	2	7	3	1
Scene3	Mean	3.60E+11	3.00E+11	1.14E+12	7.36E+03	7.20E+03	7.65E+03	9.51E+03	**6.58E+03**
	Std	3.69E+23	4.66E+23	6.67E+23	2.35E+04	2.63E+04	9.69E+04	3.06E+06	2.71E+05
	Min	7.27E+03	7.09E+03	1.15E+04	7.22E+03	6.95E+03	7.31E+03	7.18E+03	5.41E+03
	Ranking	7	6	8	3	2	4	5	1
Scene4	Mean	1.20E+11	9.30E+11	2.16E+12	1.92E+12	1.43E+04	1.53E+12	1.42E+04	**9.94E+03**
	Std	2.09E+23	8.09E+23	3.13E+23	6.00E+23	2.56E+06	2.88E+23	1.53E+06	3.02E+04
	Min	1.11E+04	1.33E+04	1.80E+12	1.62E+04	1.05E+04	2.16E+04	1.18E+04	9.76E+03
	Ranking	4	5	8	7	3	6	2	1
Scene5	Mean	8.06E+03	2.10E+11	9.00E+11	1.20E+11	7.33E+03	3.00E+10	9.57E+03	**7.18E+03**
	Std	2.03E+05	1.50E+23	2.23E+23	9.68E+22	1.17E+04	2.70E+22	8.20E+05	9.63E+04
	Min	7.63E+03	7.54E+03	1.01E+04	7.34E+03	7.15E+03	8.43E+03	8.10E+03	6.74E+03
	Ranking	3	7	8	6	2	5	4	1
Scene6	Mean	7.54E+03	4.80E+11	9.60E+11	6.00E+10	6.88E+03	9.46E+03	3.00E+10	**6.84E+03**
	Std	3.41E+05	6.00E+23	6.67E+23	1.08E+23	2.87E+04	1.34E+06	2.70E+22	6.93E+04
	Min	6.93E+03	7.41E+03	1.43E+04	6.63E+03	6.68E+03	8.05E+03	7.17E+03	6.53E+03
	Ranking	3	7	8	6	2	4	5	1
Scene7	Mean	6.91E+03	3.00E+10	1.26E+04	6.71E+03	6.63E+03	7.84E+03	7.59E+03	**6.58E+03**
	Std	2.23E+04	2.70E+22	5.32E+06	6.49E+03	3.57E+03	2.68E+05	3.12E+05	3.48E+03
	Min	6.72E+03	6.90E+03	9.01E+03	6.56E+03	6.51E+03	7.12E+03	6.84E+03	6.46E+03
	Ranking	4	8	7	3	2	6	5	1
Scene8	Mean	6.00E+10	2.40E+11	4.80E+11	6.83E+03	6.86E+03	9.42E+03	8.91E+03	**6.70E+03**
	Std	1.08E+23	3.87E+23	7.11E+23	3.67E+04	1.76E+04	1.51E+06	6.66E+05	1.96E+04
	Min	6.89E+03	7.01E+03	9.18E+03	6.65E+03	6.69E+03	7.85E+03	7.37E+03	6.51E+03
	Ranking	6	7	8	2	3	5	4	1

## Data Availability

Data is contained within the article.
